# Supplementary material for: Genome-wide analysis identifies molecular systems and 149 genetic loci associated with income
Source: Nat Commun. 2019 Dec 16;10:5741. doi: 10.1038/s41467-019-13585-5 (PMC6915786; doi:10.1038/s41467-019-13585-5)
Supplement: Supplementary file 2 — Supplementary Information [file 41467_2019_13585_MOESM2_ESM.pdf]

Supplementary Information for

Genome-wide analysis identifies molecular systems and 149 genetic loci associated with income

Hill et al.

Supplementary Methods

Publically available GWAS data on 78,308 individuals on whom intelligence was measured was meta-analysed with the data from the INTERVAL[1] study. The INTERVAL[1] study is a prospective cohort study of ~50,000 participants drawn from blood donors.[2] Recruitment occurred between 2012 and 2014 whereby blood donors aged 18 years and older consented to be recruited from the National Health Service Blood and Transplant (NHSBT) static donor centres across England. Participants are largely healthy as individuals as eligibility for donation is contingent upon being free from major disease (myocardial infarction, stroke, cancer etc.) and those who reported being unwell or having had recent illness or infection were not eligible for blood donation and so were not part of the INTERVAL sample.

Participants agreed to take part in online questionnaires that contained lifestyle and health information, including self-reported height, weight, ethnicity, current smoking status, alcohol consumption, doctor-diagnosed anemia, use of medications (hormone replacement therapy, iron supplements) and menopausal status. The INTERVAL study was approved by the Cambridge (East) Research Ethics Committee and UK Biobank was approved by the North West Multi-centre Research Ethics Committee (MREC). Informed consent was obtained from all participants.

Genotyping

DNA was extracted from whole blood using the buffy coat at LGC Genomics (UK) using a Kleargene method and samples of sufficient concentration and purity were aliquoted for shipment to Affymetrix for genotyping. Standard quality control (QC) procedures were implemented by Affymetrix during the genotyping pipeline. This included the excluding samples with a poor signal intensity (dish QC <0.82) along with samples that had a low call rate (< 0.97) based on 20,000 high quality probesets. Variants were excluded in the event that they had a call rate of <0.95, has more than three clusters, which indicates of-target measurement, had cluster statistics that were indicative of poor quality genotyping or multi-allelic variants that could not be called easily (Fisher's linear discriminant, heterozygous cluster strength, homozygote ration offset were used). A total of 48,813 participants from the INTERVAL sample were genotyped in 10 batches. Within-batch sample and variant QC was performed following standard Affymetrix QC exclusions. Non-best probesets were excluded to leave a single probeset per variant. Visual inspection of the cluster plots showed that some variants and minor allele homozygotes incorrectly called due to the presence of an extreme intensity outlier. Variants were failed from a batch in the event that there were fewer than 10 called minor allele homozygotes, the cluster plot contained at least one sample with an intensity at least twice as far from the origin as the second most extreme outlier, or if the outlying sample had an extreme polar angle (15° of > 75°) in the direction of the minor allele.

Samples that were not of European ancestry, as well as duplicate samples, were excluded prior to further QC of the variants within each batch using a set of high quality autosomal variants. These were defined as those with a minor allele frequency (MAF) >0.05, a Hardy-Weinberg equilibrium (HWE) p value of $>1\times10^{-6}$, or if there was r^2 of ≤ 0.2 between pairs of variants. Duplicate samples were identified as those where $\hat{\pi} \geq 0.9$ using Method-of-Moments IBD approach implemented in PLINK[3]. Non-Europeans were identified as those participants who had scores on PC1 or PC2 of < 0 as derived using a principal components analysis conducted on the INTERVAL samples with the 1000 Genomes major ancestry populations.[4] Variants that strongly deviated from HWE (p value $<5\times10^{-6}$), following a Fischer's exact test for low-frequency and rare variants (Those with a MAF <0.05 across all ten batches) or those with a χ^2 test for common variants were excluded from the batch. Finally, variants were also excluded from the batch in the event that they had a call rate of <0.97 (within-batch) and variants were dropped from all batches in the event that they failed in four of the ten batches due to either HWE deviation, low call rate, or failure in the Affymetrix exclusion criteria. These passing samples were then merged using the methods described by Jun et al. (2012)[5] full details can be found in Astle et al. (2016).[6] Non-autosomal and multi-allelic variants, and monomorphic variants were removed and the data were phased using SHAPEIT3 (https://jmarchini.org/shapeit3/), to phase the data in chunks of 5,000 variants with a 250 variant overlap between chunks. Imputation was conducted using a combined 1000 Genomes Phase 3-UK10K imputation panel with imputation being carried out on the Sanger Imputation Server for the 43,059 participants that remained.

Intelligence phenotype INTERVAL

A general factor of cognitive ability was derived in INTERVAL using four tests. These were the Stroop Test (a measure of attention and reaction times), Trail Making Test B (a measure of executive function), Pairs Test (a measure of episodic memory), and a Reasoning test (a measure of intelligence). These tests have been adapted from the Cardiff Cognitive Battery[7] and have been designed specifically for use in cognitive testing in epidemiology settings. Participants scores from each of these four tests was analysed using a principal components analysis where the first unrotated component was extracted. This component explained 48.0% of the variance in the test battery where each test loaded onto this single factor at 0.34-0.60. The effect of age (using fractional polynomials), sex and the effect of experiencing a stroke/TIA during the trial were controlled for using regression with the first unrotated principal component being the outcome variable. The residuals from this model were then used as the measure of intelligence for the 17,213 participants who had both genotype and phenotype data available.

Genome-wide association analysis (GWAS) in the INTERVAL sample

Individuals from INTERVAL who also took part in UK Biobank were identified using KING[8]; heterozygote concordance rate> 80% before being removed from the INTERVAL sample. A linear-mixed model was fit using BOLT-LMM[9] and adjusted for the first five principal components of ancestry. The final sample size was 17,213 individuals. Following association analysis variants where the minor allele frequency (MAF) was <0.001 and an r^2 of <0.6 were removed.

Meta-analysis of INTERVAL data

Publically available summary GWAS data on 78,308[10] participants was downloaded. These data were meta-analysed using 17,213 participants of the interval consortium using a sample size weighted meta-analysis implemented in METAL.[11] Following meta-analysis SNPs were removed if they were not present in both the INTERVAL sample and in the publically available data set on intelligence. SNPs were also excluded in the even that MAF <0.001. Z-scores were converted into beta weights using the equation, Beta <- Z-score/sqrt(2*MAF * (1-MAF) * (N+Z-score^2)), and standard errors were derived using SE <- 1/sqrt(2*MAF*(1-MAF) * (N+Z-score^2)).[12] This yielded a total of 10,895,565 SNPs following meta-analysis in a sample of 95,521 individuals.

Supplementary Note 1. Results

Functional annotation and gene based analysis of MTAG analysis of income

Functional annotation of the loci identified using MTAG proceeded using the same methods used to investigate the income phenotype from UK Biobank.[13] Firstly, it was found that 53.9% of the SNPs in the independent genomic loci were found in intronic regions and the 25.7% were found to be in intergenic regions with 14.6% being ncRNA intronic SNPs. As found in the analysis conducted without MTAG, these independent genomic loci are also predominantly found in regions of the genome involved in gene expression as indicated by 32.13% of the SNPs have an RDB score of <2 and 80.12% have a minimum chromatin state of <8 (**Supplementary Figure 2B-D** & **Supplementary Data 13**).

By examining these loci for overlap with those in GWAS Catalog we found that the loci identified using MTAG overlapped with those previously associated with intelligence (37 loci) and with education (65 loci). In addition we found that the loci linked to income were also associated with coronary artery disease and blood pressure phenotypes (7 loci) as well as with neuroticism (9 loci). Eleven loci previously linked to schizophrenia were linked to the loci associated with income and 2 loci were also linked with subcortical brain region volumes (**Supplementary Data 15**).

Gene-prioritization was also conducted on the income phenotype derived using MTAG. Here, a total of 1,317 genes were identified (**Supplementary Figure 2E** & **Supplementary Data 16**). A total of 392 genes were implicated using positional mapping with 529 implicated using eQTL analysis, with 1,006 genes identified using chromatin interaction mapping. 448 genes were implicated using two mapping strategies with 162 being identified using all three. Of the genes, 162 were identified across all three prioritization methods; 7 were of additional note as they are implicated through chromatin interactions between two independent genomic risk loci. These genes included *ORC3* on chromosome 6, a gene known with links to neuronal proliferation, which was found implicated in a cross locus interaction in the tissues of hESC, IMR90, the Liver, Mesenchymal stem cell, and Mesendoderm tissues. *ZNF589*, *NRBF2*, and *DPYD* were also implicated by all three

mapping strategies and chromatin interactions between two independent genomic risk loci in at least five tissue types (**Supplementary Data 17**).

Using a gene-based GWAS conducted in MAGMA, 312 genes attained statistical significance after controlling for multiple tests (**Supplementary Data 18**), and 101 genes were implicated by all three mapping strategies and by the MAGMA gene based GWAS.

Two gene sets were significant following correction for multiple testing neurogenesis (gene-set size = 1,337, $P = 1.67 \times 10^{-6}$) and reactome pre notch transcription and translation gene set (gene-set size = 25, $P = 3.99 \times 10^{-6}$) (**Supplementary Data 19**). The neurogenesis gene-set has been previously linked to intelligence[14] and neuroticism,[15] two phenotypes that show genetic correlations with income. This shared association between neuroticism, intelligence, and income, may therefore represent a biological system linked to all three phenotypes. It should be noted that this link between neurogenesis, neuroticism, intelligence, and income may be a case of mediated, or vertical, pleiotropy.[16]

Gene-property analysis conducted on the MTAG-income phenotype replicated the link between gene expression in the brain and association with income ($P = 1.05 \times 10^{-11}$). A link was also identified between gene expression in the pituitary gland association with income ($P = 7.72 \times 10^{-11}$). The link between expression in the testis and income whilst nominally significant ($P = 0.016$) did not withstand correction for multiple tests using the MTAG-income phenotype (**Supplementary Table 10**). This relationship between gene expression in the brain with differences in income was evident in twelve cortical tissue groupings the most significant of which were the brain cerebellar hemisphere ($P = 3.05 \times 10^{-12}$), brain cerebellum ($P = 4.36 \times 10^{-12}$), brain frontal cortex ($P = 1.03 \times 10^{-9}$), and the brain anterior cingulate cortex BA24 ($P = 4.65 \times 10^{-8}$) (**Supplementary Data 20**). A significant relationship was also found for gene expression in the early mid-prenatal developmental period and income ($P = 0.002$) (**Supplementary Table 11**) but no link was found with any of the age specific expression groupings (**Supplementary Table 12**).

These relationships between income and gene expression across the cortex are also consistent with previous findings pertaining to intelligence,[14] and so support the notion that intelligence is an intermediary phenotype between molecular genetic inheritance and individual difference in income.

Partitioned heritability conducted using stratified LDSC produced similar, but more precise, findings to the non-MTAG analysis where a significant enrichment for income was found in evolutionarily conserved regions of the genome ($P = 1.24 \times 10^{-13}$). In addition, statistically significant enrichment was also found for the histone marks of H3K9ac peaks ($P = 4.01\times 10^{-4}$), and H3K4me1 peaks ($P = 4.79 \times 10^{-4}$) as well is in this histone marks more broadly indicating a role for gene expression differences involved in income differences. Introns were also significantly enriched for income ($P = 3.26 \times 10^{-6}$).

The MTAG analysis of the continuous categories also produced similar results to the initial analysis using only household income. The continuous categories by quintile showed that common variants that were in the first three quintiles for age (i.e. the younger three groupings) were associated with a greater proportion of the heritability of income (1st quintile $P=1.00\times10^{-7}$, 2nd quintile $P=9.36\times10^{-13}$, 3rd quintile $P=3.39\times10^{-27}$) as were SNPs in the upper two quintiles for background selection greater level of background selection (4th quintile $P=5.63\times10^{-22}$, 5th quintile $P=2.59\times10^{-9}$). As found for household income, the MTAG analysis of income also showed that first three quintiles describing nucleotide diversity and the same quartiles describing the level of LD (LDD-AFR) were also significantly enriched for heritability (Nucleotide diversity, 1st quintile $P=1.23\times10^{-40}$, 2nd quintile $P=5.16\times10^{-37}$, 3rd quintile $P=9.57\times10^{-6}$, LDD-AFR, 1st quintile $P=2.43\times10^{-19}$, 2nd quintile $P=1.42\times10^{-25}$, 3rd quintile $P=2.41\times10^{-5}$) (**Supplementary Table 13**). As with household income, the enrichment found by examining the continuous annotations by quintile is consistent with the idea that negative selective pressure has acted on the partially heritable traits linked to income.

Consistent with the notion that MTAG derived a data set with associations that were specific to household income but with greater power, was the finding that cell-type specific enrichment using partitioned heritability showed the greatest level of enrichment for cell type specific groupings comes from the brain and central nervous system. Here, 29 cell types from brain and the rest of the central

nervous system were significantly enriched using the gene expression data set (**Supplementary Data 21**) compared with 24 using household income without MTAG (**Supplementary Data 10**). In addition one grouping “CD4.Positive.T.Lymphocytes”, from the blood or immune group, was found to harbour an enrichment of heritability (P=0.003)

Furthermore, using the chromatin based sets, 41 of the 42 cell groupings that were significantly enriched were drawn from the brain and the central nervous system (**Supplementary Data 22**). The remaining significant chromatin based annotation was sourced from cell types that were expressed in the digestive tissues (Esoph-Muscularis_ENTEX__H3K4me3, P = 0.002).

Three brain regions showed significant enrichment of heritability for income using the MTAG phenotype, Brain cerebellar hemisphere, $P = 3.34\times10^{-5}$, Brain cortex, P = 0.0004, and the Brain cerebellum, P = 0.001 (**Supplementary Table 14)**. Lastly, gene expression associated with the category of neuron was found to be enriched for heritability ($P=3.95\times10^{-5}$) (**Supplementary Table 15)**.

Supplementary Note 2. FAQ

FAQs regarding:

"Genome-wide analysis identifies 149 novel genetic loci associated with income"

By W. David Hill, Neil M. Davies, Stuart J. Ritchie, Nathan G. Skene, Julien Bryois, Steven Bell, Emanuele Di Angelantonio, David J. Roberts, Shen Xueyi, Gail Davies, David C. M. Liewald, David J. Porteous, Caroline Hayward, Adam S. Butterworth, Andrew M. McIntosh, Catharine R. Gale, and Ian J. Deary.
Additional queries should be directed to W. David Hill (David.Hill@ed.ac.uk).

Contents

Do your results mean that an individual's level of income is determined at birth?

What did you do in this study?

What did you find?

How could genetic differences be associated with something like household income?

What was the point of doing this research?

Have you found "the money gene"?

Okay, so there's no money *gene*, but is it fair to say you found the money *genes*?

To whom do your results apply? You said that they were from *UK* Biobank, but what about other countries?

Your paper claims a "polygenic risk score" can "predict" income. What does this mean, if genes do not determine income as you state above?

Can we now tell what someone's income is from their genes alone?

What do you mean when you talk about "intelligence"?

Aren't genetic associations with income, however small, a sign of a society that is not meritocratic?

Aren't the differences you found just due to "population stratification?

Isn't research like this going to lead to discrimination of against those who have certain genetic variants?

What are the practical applications of this research?

Do your results mean that an individual's level of income is determined at birth?

No, our results do not imply that an individual is in some way predestined to end up earning a certain amount of money. We found just a small association between genetic variation and income variation in a very large sample. That small association means that, for the most part, even people with similar genes will end up with a range of different household incomes. But there is an ever-so-slightly higher likelihood that people with particular combinations of genetic variants will earn a higher income.

The finding of genetic associations with a trait does not mean that environmental interventions cannot change them. That is, the idea of "genetic determinism" is completely false. A classic example of this is the disorder of phenylketonuria (PKU). This disorder is genetic in origin, and results in serious medical problems along with intellectual disability. However, by altering PKU patients' diets from birth, people with this condition can lead lives that are not hampered by the disease, and don't have a reduction in their cognitive abilities. Alternatively, consider eyesight. Having poorer eyesight is partly heritable, but this problem can be solved with an environmental intervention: spectacles. These examples show that, even if a trait is predominantly caused by genetic differences—and recall that income most certainly is not—this says nothing about whether it might be changed by environmental influences.

Overall, anyone who interprets the finding that "genetic factors are associated with income (or any trait)" to mean "genes determine income (or any trait), and there's nothing that can be done to change it", has fundamentally misunderstood genetic research.

What did you do in this study?

We performed a "genome-wide association study", or GWAS (pronounced "jee-was"). A GWAS is a type of study that searches for genetic variants that are linked to some outcome: they've been extensively used to find the genetic links to health conditions like cardiovascular disease and schizophrenia, and traits like height and weight. Our particular GWAS examined the outcome of household income, which might sound surprising since household income doesn't—at least at first—appear to be a condition or a trait like those we just listed. We'll explain this choice in more detail below.

The genetic variants we just mentioned are called Single-Nucleotide Polymorphisms, or "SNPs" (pronounced "snips"). These are points on the DNA—many millions of them—where people tend to differ in which nucleotide (which one of the four chemical "letters" of DNA: A, C, T, and G) is present. For example, 25% of people might have an "A" at that point on their DNA, whereas the remaining 75% have a "G". This is the most common, though far from the only, way in which people differ in their DNA. A GWAS study lines up all the SNPs a person has and tests the extent to which each one is linked to the outcome of interest. In the case of height, each SNP might contribute a tiny fraction towards explaining, why some people are taller than others – contributing to bone length, say, or growth hormone production.

We did our GWAS in DNA data from 286,301 people from across the United Kingdom, aged 39-73 years. These were participants in the large-scale UK Biobank study. They had provided

a large amount of information on their health and wellbeing, as well as a DNA sample. Our GWAS tested whether differences in household income were related to differences in SNPs. The first thing we did was the standard GWAS methodology as described above: we examined if people whose household income was higher, were more likely to have particular versions of each SNP in their genome.

Our second analysis was to look at whether people who were generally more similar across all their SNPs were closer to each other in their income – this is one way of looking at the "heritability" of income. Heritability is the proportion of variation in the trait that is explained by all the genetic variants, in this case SNPs.

Next, we combined our genetic data with information from other, previous studies on what genes are actually *doing*: how they are "expressed", where specifically in the DNA they are located, and what biological functions they are thought to have. By doing so, we could try to understand the likely biological processes that any income-linked genetic variants were involved in. Finally, we looked at one likely psychological candidate for the link between DNA and income: intelligence, which is sometimes called cognitive ability or cognitive function. Our previous work had hinted that the reason DNA variants are associated with income in the first place is that—in part—they might predispose people to higher intelligence, which helps them attain higher incomes. Here, we used some more complex genetic statistical analyses that allowed us to look at which effects were *causal* – specifically, we checked whether part of the chain of causality was *genes->intelligence->income*. Even if we could find evidence to support this, we acknowledge that the chain would be far longer and more complicated than this, and would also include pathways that go from *income->intelligence* without any genes being involved. See below for more on what we mean by "intelligence".

What did you find?

As we expected, the majority of the reasons people differed in their level of household income were *not* genetic. Our view is that those reasons are likely to be "environmental" – by this we mean, that the likely reasons are to do with anything that is not genetic: these include social conditions, economic policies like tax and benefit rates, pure luck, and so on. However, we *did* find a small association between genetic variation and variation in household income. We shall speculate as to why this might be in our answer to the next question. First, we'll describe the results in more detail.

We found that variation across all the SNPs measured in the current study from the UK Biobank sample accounted for 7.4% of the variation in household income. This is a small amount and other studies that have used methods to capture less common genetic variation have found heritability estimates for income between 40-50%.[17] Therefore it is likely that our estimate, based only on common genetic variation, is an underestimation of the total genetic effect.

The variation in income that is not due to genetic effects is likely due to environmental factors, as well as errors of measurement (for example, people often make mistakes filling in questionnaires, including questionnaires about income, and this creates some "noise" in the measure). Therefore, as we noted above, a large proportion of people's differences in household income is likely to be environmental in origin, according to our results.

We identified 30 regions of the genome (termed “loci”) that were associated with individual differences in household income (**Figure 3**). The SNPs within these loci showed evidence of being involved in gene-expression differences, and we identified that genes expressed in the brain and the synapse were associated with income (**Figure 4** & **Figure 5**). We also found evidence that genes involved in neurogenesis, the process by which new neurons are formed, were associated with household income (**Supplementary Note 1. Results**). Whereas we were able to detect these differences each individual SNP had only a miniscule association with household income, even though each of them was statistically significant.

We found that the genetic differences that were associated with higher household income (i.e. those that accounted for that 7.4% of household income variation) overlapped with the genetic differences that were linked to longer life, health, wellbeing, and intelligence (**Figure 6A, B & C**). We found that the genetic variations that were linked to higher household income overlapped with the genetic differences that were associated with a lower risk of developing schizophrenia, ADHD, coronary artery disease, and feelings of tiredness and fatigue (**Figure 6A, B & C**).

When we used the causal analysis mentioned above (a technique called Mendelian randomisation), we found evidence that being more intelligent was causally related to having a higher income.

How could genetic differences be associated with something like household income?

At first, it seems confusing that genetic differences might be associated with household income. A UK person’s income is a social variable, influenced by factors such as the socioeconomic position of their parents, the part of the country they happen to live in, how the economy is doing, politics and social policies, health and the physical environment, education, other factors, and, of course, luck.

All of the factors we just listed and more are likely to play a role in people’s income levels – the reasons some people earn more money than others are complex. But on top of these, there are some individual traits and characteristics that might make a person more or less likely to earn a higher income. They might be more highly motivated and conscientious at work, for example, or they might be more intelligent, be less susceptible to illness, or simply more interested in the kinds of jobs that lead to higher wages. Some or all of these, along with other psychological and health factors, might be associated, to some extent, with genetic differences. Those genetic differences, which might have effects in the brain or in other parts of the body that are important for maintaining one’s health, might affect income via their effects on these other, “intermediate” traits, especially if these genetic variants have effects in the brain or in other parts of the body that are important for maintaining one’s health. In our study, we particularly focus on intelligence (or cognitive ability) as one of these potential factors, and we find it might account for some of the gene-income link. But this is not to gainsay the importance of all those other factors.

What was the point of doing this research?

The prompt for the study was our interest in health inequalities and how to ameliorate them. It is well known that people with a lower income are at greater risk of poorer health than

those from more advantaged social backgrounds. Research into the reasons for these health differences investigates all the possible explanations for this link, be they environmental, genetic, or otherwise. For example, previous studies have shown that many common health problems are partly due to the genes that people inherit. A small part of the variation in social factors such as income also appears to be modestly associated with differences in their genes. Thus, one of the reasons genes might be related to income is that the genes cause health problems, which cause you to receive a lower income. We wanted to investigate whether the associations between income and health might, at least partly, be accounted for by people's genetic differences that are associated with both income and health. If politicians or others wish to make effective policies to reduce the health inequalities between people of different social backgrounds, they need to know as much as possible about the mechanisms that produce these health inequalities. There is a great deal of extremely important research on the environmental factors that cause health and income inequalities. Our results make a small start at understanding the biological factors – which, to emphasize again, do not gainsay the existence or importance of environmental factors, and are themselves only a small part of the story of why people differ in their household income.

Have you found "the money gene"?

No, we have not found 'the money gene', or 'the gene for income'. Income variation is a complex social measure, and it has many influences. Some potential influences on income—such as some illnesses and some personal traits—are themselves partly heritable. It is possible that some genetic associations with such factors (health, personal traits etc.) are also picked up in a GWAS study of income. Therefore, our GWAS results should not be interpreted as indicating that there are close links between genetic variations and income differences.

Moreover, there was no 'gene for income' in the sense that there was no large association between any individual genetic variant and differences in income. Genetic associations with factors such as household income (or height, or intelligence, or many common illnesses) are composed of many thousands of genetic variants, each with a tiny association – they all add up to form the overall genetic contribution to a trait or condition.

Okay, so there's no money *gene*, but is it fair to say you found the money *genes*?

No, we did not find 'money genes'. The reason for this is that we don't think there can be direct genetic effects that act on social variables such as income; income and other measures of SEP are themselves the products of multiple factors and traits. Therefore, whereas the genes identified are associated with differences in income, they are also associated with other traits that may be causally related to income in Great Britain today.

To whom do your results apply? You said that they were from *UK* Biobank, but what about other countries?

Our findings were made in the context of Great Britain (that is, the participants were from England, Scotland, and Wales) in the modern era (that is, the participants filled in their questionnaire on their household income at some point within the past approximately ten

years). In addition, our analysis was restricted to people who described their ethnic background as White. It will be interesting to test the extent to which the income-linked genetic variants we've found apply to people of different backgrounds, and those from different countries, cultures, and time periods, but for now, our data cannot speak to them.

In addition, we should emphasize that the results we have found apply to what *is* currently the case Great Britain – not what *could* be the case, under different policies or economic circumstances. For example, a government could insist that everyone had the same income, at which point the genetic contribution to income differences would necessarily fall to zero. We do not attach any moral value to our findings: we are not arguing that people with "better genes" (whatever that would mean) are somehow getting their just deserts. Our findings are purely descriptive, and, although we note elsewhere that they can be used to inform policies that attempt to meliorate social inequalities, we do not take any view on what those policies should be.

Your paper claims a "polygenic risk score" can "predict" income. What does this mean, if genes do not determine income as you state above?

Another finding in our study was that we can take the results of our GWAS and create a so-called "polygenic risk score" in a new, independent sample of people. This is essentially a summary of how many genetic variants a person has that give them a higher likelihood of a certain outcome – in our case, higher income. We found that 2.5% of the income differences in a new sample could be accounted for by this polygenic score.

In statistics, the word "prediction" is often used differently from its common usage. Prediction would commonly refer to a statement regarding the future; however, in statistics, prediction can be used to transfer knowledge from one sample to another. In our study, we used knowledge of the genetic variants linked to income in UK Biobank (sample 1) to test if these same variants were linked to income in another sample, Generation Scotland (sample 2). In doing so, knowledge of which genes were linked with income was transferred across these two samples. By testing whether the same genetic variants were associated with income in these two samples, we were able to link 2.5% of variation in income in Generation Scotland to the genetic effects identified in UK Biobank. Or, put another way, using the knowledge of which genetic variants were linked to income in UK Biobank, we were able to predict 2.5% of people's income differences in Generation Scotland based solely on their DNA.

Can we now tell what someone's income is from their genes alone?

No, polygenic risk scores used in our report cannot make accurate predictions of an individual person's household income. The predictive ability of a polygenic risk score that only accounts for 2.5% of the differences in an outcome is extremely low, and this is even worse when applied to individual people. To put this another way, the score was *not* informative as to the remaining 97.5% of the variation in household income in the Great Britain today. As seen in **Figure 7A,** many of the individuals with a household income of <£10,000 had a greater polygenic risk score for income than those earning >£70,000. This is

strong evidence against the idea that the genes we identify here "determine" a person's income (and, as we noted above, we would argue that this logic applies to essentially all genetic influences).

Aren't genetic associations with income, however small, a sign of a society that is not meritocratic?

Although such questions are unavoidable given that income was the main focus of the research, we did not directly address political, ethical, and moral issues like this one in our study. How one defines "meritocracy", and the evidence one accepts to show the presence or lack of meritocracy in a society, will depend on one's political priors, and we are not equipped or qualified to provide a discussion of political theory in this study or this FAQ document. However, it is important to note that our findings are descriptive, not prescriptive: none of the findings we describe should be read as saying that anyone "deserves" a particular income, since of course one does not "deserve" any particular set of genes one is given at conception.

Nevertheless, for the sake of argument, it could be said that no, our results do not necessarily indicate a lack of meritocracy. Genetic associations with outcomes such as income can be an indicator of some degree of meritocracy. Imagine that we had found no genetic associations with income. What might this mean? It would indicate that the genetic variants associated with, say, higher intelligence, or greater conscientiousness—factors that most meritocrats might wish to reward—were in fact *not* associated with income. Therefore, our finding of a modest genetic association with income could be viewed as an indicator of the "meritocratic" notion that, at least within the population studied here, that an individual's level of ability or industriousness, intelligence/personality/health—partly influenced by genetic effects—is associated, at least to some extent, with a higher income. Overall, though, merely knowing that income is related to genetic differences is just one tiny factor in answering the larger, and much more complicated, question of whether or not a society is meritocratic.

What do you mean when you talk about "intelligence"?

As discussed above, we found evidence in our study that intelligence was one of the factors that may be a causal factor in attaining a higher income. Indeed, one of the advances that our study makes is that it provides evidence that intelligence is not just correlated with income, but has a causal effect on income – that is to say, being more intelligent causes—to some extent, and on average—higher income. But what do we mean by "intelligence"? Here, we simply defined intelligence as a person's score on a cognitive test, because that was how it was defined in the previous GWAS whose data on intelligence that we use in our analysis.

Whether this definition matches with how people broadly define "intelligence" in society—or your own personal definition of the term—is certainly up for debate. Intelligence-type tests certainly miss important aspects of human ability, and they are far from the be-all and end-all of understanding human psychological differences. But what matters for our particular analysis is that people's scores on these tests appeared to be causally related to income, indicating that the kind of skills that the tests require—reasoning abilities, mental speed, memory, and so on—are helpful in gaining socioeconomic status, at least in the context we studied (British people in the modern era). As we noted above, though, this doesn't gainsay

other influences on income that have nothing to do with intelligence, and it doesn't gainsay effects of income on intelligence, rather than the other way around. All these explanations aren't mutually exclusive, and it's our view that investigating them all should be of interest to anyone who wants to understand societal differences.

Aren't the differences you found just due to "population stratification"?

This question is a rather technical one, but it is nonetheless important. Put as simply as possible, if there is also a difference in income between particular sub-groups of the population (for example, people in the north having lower incomes than those in the south of the UK) and if they also happen to have slightly different genes due to chance (a well-known biological process called "genetic drift" can mean that this is the case), then this could lead to a spurious association between a variant and income. Put more technically, population stratification describes the existence of a systematic difference in allele frequencies (that is, the counts of specific types of each SNP) between population sub-groups within a larger population. The frequency of different variants of a gene may diverge across a population over the generations due to genetic drift; this can lead to genetic differences between subgroups within a population whereby, in one of the subgroups a particular allele may be very rare but for another subgroup, within the same population, this allele is much more common. This can be very misleading, and it was therefore important in our study to take steps to minimize, and to test for, these population stratification effects.

We used two methods to limit the effect that population stratification had on our results. First, we ensured that all our participants originated from a similar ancestral background. In this case, all our participants were white British individuals. Second, we controlled for the degree to which genetic clustering occurred by including 40 principal components derived from the genotyped DNA-SNP data in our analysis. By doing this, we sought to break the association between SNPs and a phenotype that could have been caused by population stratification. Finally, we tested for population stratification using a method called linkage disequilibrium score regression (LDSC)[18]. LDSC is used to test whether the results of a GWAS are due to population stratification or are due to many variants each associated with a small effect (what is termed polygenicity). In our study we used LDSC regression, and the results indicated that over 92% of the signal we identified in our GWAS on income was due to a large number of genetic variants each exerting a small effect (i.e., a polygenic effect) rather than population stratification.

Isn't research like this going to lead to discrimination of against those who have certain genetic variants?

Unfortunately, a lot of scientific research has the potential to be misused or even abused. Even a study aiming to identify genetic variants linked with Alzheimer's disease could be abused to discriminate against those who have variants that predispose them towards this illness; for example, such carriers might be subject to an increased cost of health insurance.

Importantly, research into the genetic basis of traits such as income can also facilitate a reduction in discrimination, precisely because the effect sizes of genetic effects that act on these traits becomes a known variable. In the current study, we found that genetic effects

accounted for around only 7.5% of income differences in Great Britain today and that these genetic effects overlapped with intelligence as well as physical and mental health. This would mean that ignorant and incorrect arguments that would be used to discriminate against those with low income on the basis that inequality is built into our DNA[19] are empirically falsified by our study for two reasons. First, the genetic variants tested account for only 7.5% of income in this population sample. Secondly, as seen in our **Figure 7A**, those with a high polygenic score for income often earn less that those with a low polygenic score for income; that's what happens when there are only small associations between variables.

We emphasize the fact that we are describing the world as it is, not how it could be; we are also describing results that apply to a specific context, and not others. Under different circumstances, the genetic effects could be smaller or larger, and so could people's incomes. Regardless of any effects of genetics, those circumstances are, to a very substantial extent, under our and others' control.

What are the practical applications of this research?

At present, there are none.

We do not test any practical applications of our genetic knowledge in this study, and nor do we advocate for any. This is basic (as opposed to applied) science, finding links and causes among interesting and important variables, and building a picture of how genes relate to different phenotypes. The study does not include an intervention to attempt to improve those phenotypes, or anything close to it. Should this research translate towards anything practical, it will be a long time in the future, and after a great deal more research, as well as careful and ethical testing of every step along the way.

Supplementary Table 1. MAGMA gene-property analysis for household income.

Tissue group	Beta	Standard error of Beta	P value	Significant
Brain	0.034	0.008	1.31×10^{-5}	1
Testis	0.023	0.007	3.17×10^{-4}	1
Pituitary	0.029	0.011	0.002	0
Ovary	0.028	0.014	0.022	0
Thyroid	0.017	0.015	0.137	0
Uterus	0.018	0.017	0.146	0
Pancreas	0.009	0.011	0.200	0
Cervix Uteri	0.012	0.025	0.318	0
Muscle	0.003	0.008	0.370	0
Prostate	0.004	0.021	0.420	0
Stomach	0.003	0.019	0.441	0
Blood	0.001	0.007	0.466	0
Adrenal Gland	0.000	0.013	0.507	0
Nerve	-0.003	0.015	0.575	0
Blood Vessel	-0.006	0.017	0.630	0
Colon	-0.009	0.025	0.644	0
Fallopian Tube	-0.010	0.021	0.680	0
Small Intestine	-0.013	0.014	0.835	0
Bladder	-0.023	0.022	0.858	0
Esophagus	-0.034	0.028	0.889	0
Vagina	-0.024	0.019	0.895	0
Skin	-0.019	0.014	0.915	0
Heart	-0.017	0.012	0.922	0
Liver	-0.011	0.008	0.925	0
Kidney	-0.019	0.013	0.934	0
Breast	-0.046	0.026	0.961	0
Lung	-0.028	0.015	0.970	0
Adipose Tissue	-0.038	0.020	0.972	0
Spleen	-0.019	0.010	0.975	0
Salivary Gland	-0.031	0.015	0.981	0

Gene sets that withstood Bonferroni correction are highlighted in bold. 30 general tissue types were created using gene expression data based on GTEx RNA-seq data. Significant = 1, indicates tissue groupings that withstood Bonferroni correction.

Supplementary Table 2. MAGMA gene-property analysis for household income aiming to identify the cell types that are associated with differences in income.

Cell Type	P Enrichment one Tailed	Significant
Medium Spiny Neurons	7.67×10^{-5}	1
Serotonergic Neurons	0.002	1
Embryonic Dopaminergic Neurons	0.004	0
Somatosensory Cortex Pyramidal Neurons	0.009	0
Cortical Interneurons	0.009	0
Neuronal Progenitor	0.013	0
Striatal Interneuron	0.023	0
Oligodendrocyte Precursor	0.028	0
Hypothalamic GABAergic Neurons	0.037	0
Pyramidal (CA1)	0.037	0
Dopaminergic Neuroblast	0.045	0
Adult Dopaminergic Neurons	0.057	0
Embryonic Midbrain Nucleus Neurons	0.061	0
Neuroblasts	0.063	0
Embryonic GABAergic Neurons	0.065	0
Oxytocin / Vasopressin Neurons	0.087	0
Hypothalamic Dopaminergic Neurons	0.123	0
Radial Glia Like	0.259	0
Oligodendrocytes	0.388	0
Hypoth. Glutamatergic Neurons	0.510	0
Endothelial-Mural	0.545	0
Microglia	0.596	0
Vascular Leptomeningeal Cells	0.604	0
Astrocytes / Ependymal	0.657	0

Significant = 1, indicates gene sets that withstood Bonferroni correction.

Supplementary Table 3. MAGMA gene-property analysis for household income.

General developmental stages of brain samples	Beta	SE	P
Early prenatal	0.029	0.013	0.014
Early mid-prenatal	0.038	0.016	0.008
Late mid-prenatal	0.032	0.022	0.066
Late prenatal	-0.017	0.027	0.739
Early infancy	-0.092	0.071	0.904
Late infancy	-0.007	0.041	0.566
Early childhood	-0.046	0.022	0.981
Late childhood	-0.070	0.034	0.980
Adolescence	-0.023	0.038	0.722
Young adulthood	-0.004	0.034	0.549
Middle adulthood	-0.022	0.025	0.816

A total of 11 general development stages were created using gene expression data based on the BrainSpan data (http://www.brainspan.org/static/download).

Supplementary Table 4. MAGMA gene-property analysis for household income.

Ages of brain samples	Beta	SE	P
8 PCW	0.0001	0.0002	0.371
9 PCW	0.0002	0.0003	0.250
12 PCW	0.0001	0.0003	0.330
13 PCW	0.0001	0.0003	0.372
16 PCW	0.0002	0.0003	0.273
17 PCW	0.0002	0.0004	0.270
19 PCW	0.0001	0.0002	0.383
21 PCW	0.0002	0.0004	0.317
24 PCW	0.0002	0.0003	0.284
26 PCW	-0.0004	0.0007	0.742
37 PCW	0.0001	0.0003	0.366
4 Months	-0.0003	0.0008	0.664
10 Months	-0.0001	0.0004	0.595
1 Year	0.0003	0.0005	0.274
2 Years	-0.0004	0.0003	0.913
3 Years	-0.0003	0.0003	0.835
4 Years	-0.0002	0.0003	0.761
8 Years	-0.0004	0.0004	0.827
11 Years	0.0000	0.0005	0.468
13 Years	0.0001	0.0005	0.412
15 Years	0.0003	0.0005	0.300
18 Years	-0.0003	0.0004	0.791
19 Years	0.0000	0.0004	0.469
21 Years	0.0001	0.0005	0.454
23 Years	-0.0003	0.0005	0.741
30 Years	0.0000	0.0004	0.472
36 Years	0.0001	0.0005	0.446
37 Years	0.0002	0.0005	0.384
40 Years	-0.0003	0.0004	0.798

Gene sets that withstood Bonferroni correction are highlighted in bold. 29 age groups were created using gene expression data based on the BrainSpan data (http://www.brainspan.org/static/download). PCW, post conception weeks.

Supplementary Table 5. Analysis of continuous annotations by quintile for household income.

Grouping	Quintile	Enrichment	Enrichment SE	Enrichment P-value	Significant
Predicted Allele Age	1st	1.415	0.108	$2.57{\times}10^{-4}$	1
Predicted Allele Age	2nd	1.216	0.040	$3.33{\times}10^{-7}$	1
Predicted Allele Age	3rd	1.067	0.009	$6.91{\times}10^{-16}$	1
Predicted Allele Age	4th	0.884	0.044	0.009	0
Predicted Allele Age	5th	0.418	0.103	$1.21{\times}10^{-7}$	0
Background Selection	1st	0.823	0.040	$1.58{\times}10^{-6}$	0
Background Selection	2nd	0.906	0.021	$2.57{\times}10^{-6}$	0
Background Selection	3rd	1.015	0.015	0.299	0
Background Selection	4th	1.089	0.018	$9.81{\times}10^{-8}$	1
Background Selection	5th	1.171	0.053	0.001	1
Recombination rate	1st	0.949	0.043	0.251	0
Recombination rate	2nd	1.022	0.034	0.519	0
Recombination rate	3rd	1.045	0.033	0.165	0
Recombination rate	4th	1.086	0.024	$1.99{\times}10^{-4}$	1
Recombination rate	5th	0.899	0.101	0.306	0
LDD AFR	1st	1.479	0.058	$5.38{\times}10^{-12}$	1
LDD AFR	2nd	1.200	0.020	$7.36{\times}10^{-16}$	1
LDD AFR	3rd	1.033	0.011	0.002	1
LDD AFR	4th	0.841	0.025	$3.30{\times}10^{-8}$	0
LDD AFR	5th	0.446	0.051	$2.62{\times}10^{-18}$	0
Nucleotide Diversity	1st	1.456	0.046	$2.47{\times}10^{-23}$	1
Nucleotide Diversity	2nd	1.219	0.023	$3.79{\times}10^{-20}$	1
Nucleotide Diversity	3rd	1.032	0.010	0.003	1
Nucleotide Diversity	4th	0.850	0.017	$3.40{\times}10^{-20}$	0
Nucleotide Diversity	5th	0.410	0.063	$2.97{\times}10^{-19}$	0
CpG Content	1st	0.840	0.034	$1.52{\times}10^{-5}$	0
CpG Content	2nd	0.991	0.024	0.709	0
CpG Content	3rd	0.998	0.019	0.906	0
CpG Content	4th	0.939	0.023	0.007	0
CpG Content	5th	1.232	0.055	$4.32{\times}10^{-5}$	1

Statistical significance was derived using a Bonferroni correction ($\alpha = 0.01$) within each quintile where 1 = Yes; 0 = No.

Supplementary Table 6. Showing the enrichment of household income for 13 brain annotations based on the GTEx database.

Brain Region	Coefficient	Coefficient SE	Coefficient P-value	Significant
Brain Cortex	3.38×10^{-9}	1.05×10^{-9}	0.001	1
Brain Frontal Cortex (BA9)	2.21×10^{-9}	1.02×10^{-9}	0.015	0
Brain Cerebellar Hemisphere	2.52×10^{-9}	1.25×10^{-9}	0.022	0
Brain Nucleus accumbens (basal ganglia)	2.05×10^{-9}	1.45×10^{-9}	0.078	0
Brain Anterior cingulate cortex (BA24)	1.45×10^{-9}	1.03×10^{-9}	0.081	0
Brain Cerebellum	1.51×10^{-9}	1.30×10^{-9}	0.122	0
Brain Putamen (basal ganglia)	3.96×10^{-10}	1.53×10^{-9}	0.398	0
Brain Amygdala	3.18×10^{-12}	1.24×10^{-9}	0.499	0
Brain Caudate (basal ganglia)	-2.14×10^{-10}	1.45×10^{-9}	0.559	0
Brain Spinal cord (cervical c-1)	-5.41×10^{-10}	1.07×10^{-9}	0.693	0
Brain Hypothalamus	-2.72×10^{-9}	1.01×10^{-9}	0.996	0
Brain Substantia nigra	-2.76×10^{-9}	1.01×10^{-9}	0.997	0
Brain Hippocampus	-3.25×10^{-9}	9.42×10^{-10}	1.000	0

Multiple comparisons were controlled for using a Bonferroni correction across the 13 annotations (α = 0.003846154) where 1 = Yes; 0 = No.

Supplementary Table 7. Showing the enrichment of household income for 3 brain cell types taken from Cahoy et al. (2008).

Brain Cell Type	Coefficient	Coefficient SE	Coefficient P-value	Significant
Neuron	$4.19{\times}10^{-9}$	$1.30{\times}10^{-9}$	0.001	1
Astrocyte	$-1.26{\times}10^{-10}$	$1.29{\times}10^{-9}$	0.539	0
Oligodendrocyte	$-9.05{\times}10^{-10}$	$1.33{\times}10^{-9}$	0.752	0

Multiple comparisons were controlled for using a Bonferroni correction across the 3 annotations (α = 0.01666667) where 1 = Yes; 0 = No.

Supplementary Table 8. The results of the Mendelian Randomisation analysis performed on intelligence and income using 19 SNPs from restricted set of 95k individuals.

Section A: Causal effect of intelligence on income (19 SNPs from restricted set of 95k individuals).

	N SNPs	Beta	SE	P
MR Egger	19	-0.079	0.222	0.73
Weighted median	19	0.193	0.040	1.86×10^{-6}
Inverse variance weighted	19	0.213	0.063	7.63×10^{-4}
Simple mode	19	0.172	0.067	0.02
Weighted mode	19	0.161	0.061	0.02

Section B: Heterogeneity statistics

Method	Q	Q df	P
Maximum likelihood	120.0	18	4.28×10^{-17}
MR Egger	120.5	17	1.22×10^{-17}
Inverse variance weighted	133.8	18	1.00×10^{-19}
IVW radial	120.5	18	3.34×10^{-17}

Section C: Egger Intercept for effect of intelligence on income

	Intercept	SE	P
Egger intercept	0.010	0.007	0.189

Scale is 1 SD change in intelligence on a unit change in income (Income was defined as a five point category of 1,2,3,4,5. See Methods for full details of phenotype definition).

Supplementary Table 9. Polygenic risk score analysis results.

Base Data	Threshold	Beta	S.E.	r^2	P-value
Income UK Biobank	0.01	0.111	0.012	0.0122	$< 2.2\times10^{-16}$
	0.05	0.132	0.012	0.0175	$< 2.2\times10^{-16}$
	**0.1**	**0.143**	**0.012**	**0.0204**	$\mathbf{< 2.2\times10^{-16}}$
	0.5	0.135	0.012	0.0183	$< 2.2\times10^{-16}$
	1	0.137	0.012	0.0189	$< 2.2\times10^{-16}$
MTAG Income					
	0.01	0.133	0.012	0.0176	$< 2.2\times10^{-16}$
	**0.05**	**0.157**	**0.012**	**0.0247**	$\mathbf{< 2.2\times10^{-16}}$
	0.1	0.153	0.012	0.0233	$< 2.2\times10^{-16}$
	0.5	0.151	0.012	0.0228	$< 2.2\times10^{-16}$
	1	0.153	0.012	0.0234	$< 2.2\times10^{-16}$

Phenotypic variation in income was predicted in the Generation Scotland: Scottish Family Health Study. The p-value cut off that generated the predictor that explained the most variance is shown in bold.

Supplementary Table 10. MAGMA gene-property analysis for the MTAG analysis of household income.

Tissue group	Beta	Standard error of Beta	P value	Significant
Brain	0.062	0.009	1.05×10^{-11}	1
Pituitary	0.047	0.012	6.72×10^{-5}	1
Testis	0.017	0.008	0.016	0
Ovary	0.031	0.016	0.031	0
Muscle	0.008	0.009	0.177	0
Nerve	0.011	0.018	0.262	0
Uterus	0.013	0.022	0.268	0
Pancreas	0.007	0.012	0.269	0
Adrenal Gland	0.005	0.016	0.367	0
Heart	0.003	0.014	0.426	0
Blood Vessel	0.003	0.021	0.447	0
Colon	0.001	0.032	0.487	0
Cervix Uteri	-0.007	0.032	0.592	0
Blood	-0.002	0.008	0.600	0
Thyroid	-0.006	0.019	0.617	0
Prostate	-0.009	0.027	0.634	0
Bladder	-0.020	0.027	0.771	0
Skin	-0.013	0.017	0.780	0
Esophagus	-0.031	0.036	0.804	0
Stomach	-0.021	0.023	0.817	0
Fallopian Tube	-0.030	0.027	0.863	0
Vagina	-0.026	0.024	0.869	0
Breast	-0.044	0.033	0.906	0
Salivary Gland	-0.030	0.018	0.951	0
Adipose Tissue	-0.041	0.025	0.951	0
Spleen	-0.020	0.011	0.957	0
Small Intestine	-0.030	0.017	0.964	0
Kidney	-0.033	0.015	0.988	0
Liver	-0.020	0.009	0.989	0
Lung	-0.044	0.018	0.993	0

A total of 30 general tissue types were created using gene expression data based on GTEx RNA-seq data. Significant = 1, indicates tissue groupings that withstood Bonferroni correction.

Supplementary Table 11. MAGMA gene-property analysis for the MTAG analysis of household income.

General developmental stages of brain samples	Beta	SE	P	Significant
Early prenatal	0.037	0.016	0.011	0
Early mid-prenatal	0.058	0.019	0.002	1
Late mid-prenatal	0.058	0.027	0.017	0
Late prenatal	-0.057	0.035	0.948	0
Early infancy	-0.140	0.100	0.920	0
Late infancy	0.020	0.056	0.364	0
Early childhood	-0.078	0.028	0.998	0
Late childhood	-0.059	0.045	0.903	0
Adolescence	-0.039	0.051	0.779	0
Young adulthood	-0.002	0.045	0.518	0
Middle adulthood	-0.037	0.032	0.872	0

A total of 11 general development stages were created using gene expression data based on the BrainSpan data (http://www.brainspan.org/static/download). Significant = 1, indicates groupings that withstood Bonferroni correction.

Supplementary Table 12. MAGMA gene-property analysis for the MTAG analysis of household income.

Ages of brain samples	Beta	SE	P
8 pcw	7.53×10^{-5}	0.0003	0.391
9 pcw	0.0003	0.0003	0.144
12 pcw	0.0003	0.0003	0.130
13 pcw	0.0002	0.0003	0.215
16 pcw	0.0003	0.0003	0.165
17 pcw	0.0003	0.0004	0.182
19 pcw	-5.36×10^{-5}	0.0002	0.592
21 pcw	0.0005	0.0004	0.094
24 pcw	0.0003	0.0003	0.170
26 pcw	-0.0010	0.0007	0.917
37 pcw	-9.67×10^{-6}	0.0003	0.511
4 mos	-1.72×10^{-5}	0.0008	0.509
10 mos	0.0002	0.0005	0.350
1 yrs	0.0009	0.0006	0.067
2 yrs	-0.0008	0.0003	0.996
3 yrs	-0.0006	0.0004	0.959
4 yrs	-0.0005	0.0003	0.931
8 yrs	-0.0003	0.0004	0.760
11 yrs	9.64×10^{-6}	0.0005	0.493
13 yrs	3.39×10^{-5}	0.0005	0.475
15 yrs	0.0005	0.0005	0.196
18 yrs	-0.0007	0.0004	0.967
19 yrs	0.0002	0.0005	0.374
21 yrs	0.0004	0.0005	0.241
23 yrs	-0.0004	0.0005	0.802
30 yrs	-5.26×10^{-5}	0.0004	0.555
36 yrs	0.0002	0.0006	0.364
37 yrs	0.0003	0.0006	0.321
40 yrs	-0.0006	0.0004	0.932

A total of 29 age groups were created using gene expression data based on the BrainSpan data (http://www.brainspan.org/static/download). Groupings that withstood Bonferroni are highlighted in bold.

Supplementary Table 13. Analysis of continuous annotations by quintile using the MTAG analysis of income.

Grouping	Quintile	Enrichment	Enrichment SE	Enrichment P-value	Significant
Predicted Allele Age	1st	1.475	0.084	1.00×10^{-7}	1
Predicted Allele Age	2nd	1.243	0.031	9.36×10^{-13}	1
Predicted Allele Age	3rd	1.069	0.007	3.39×10^{-27}	1
Predicted Allele Age	4th	0.861	0.034	8.52×10^{-5}	0
Predicted Allele Age	5th	0.352	0.080	1.20×10^{-13}	0
Background Selection	1st	0.741	0.029	2.70×10^{-20}	0
Background Selection	2nd	0.877	0.017	8.91×10^{-14}	0
Background Selection	3rd	1.014	0.012	0.245	0
Background Selection	4th	1.123	0.013	5.63×10^{-22}	1
Background Selection	5th	1.252	0.044	2.59×10^{-9}	1
Recombination rate	1st	1.001	0.033	0.980	0
Recombination rate	2nd	1.054	0.022	0.012	0
Recombination rate	3rd	1.062	0.023	0.005	1
Recombination rate	4th	1.083	0.019	1.24×10^{-5}	1
Recombination rate	5th	0.804	0.064	0.001	0
LDD AFR	1st	1.454	0.044	2.43×10^{-19}	1
LDD AFR	2nd	1.187	0.015	1.42×10^{-25}	1
LDD AFR	3rd	1.035	0.009	2.41×10^{-5}	1
LDD AFR	4th	0.853	0.018	4.11×10^{-13}	0
LDD AFR	5th	0.472	0.041	3.97×10^{-29}	0
Nucleotide Diversity	1st	1.509	0.037	1.23×10^{-40}	1
Nucleotide Diversity	2nd	1.239	0.018	5.16×10^{-37}	1
Nucleotide Diversity	3rd	1.036	0.008	9.57×10^{-6}	1
Nucleotide Diversity	4th	0.826	0.013	1.99×10^{-36}	0
Nucleotide Diversity	5th	0.354	0.049	8.66×10^{-36}	0
CpG Content	1st	0.857	0.026	1.74×10^{-7}	0
CpG Content	2nd	0.994	0.018	0.744	0
CpG Content	3rd	1.006	0.015	0.700	0
CpG Content	4th	0.939	0.019	0.001	0
CpG Content	5th	1.205	0.040	3.21×10^{-7}	1

Statistical significance was derived using a Bonferroni correction ($\alpha = 0.01$) within each quintile where 1 = Yes; 0 = No.

Supplementary Table 14. Showing the enrichment of the MTAG analysis of household income for 13 brain annotations based on the GTEx database.

Brain Region	Coefficient	Coefficient SE	Coefficient P-value	Significant
Brain Cerebellar Hemisphere	4.47×10^{-9}	1.12×10^{-9}	3.34×10^{-5}	1
Brain Cortex	3.00×10^{-9}	9.06×10^{-10}	4.72×10^{-4}	1
Brain Cerebellum	3.45×10^{-9}	1.14×10^{-9}	0.001	1
Brain Frontal Cortex (BA9)	1.90×10^{-9}	8.00×10^{-10}	0.009	0
Brain Anterior cingulate cortex (BA24)	1.57×10^{-9}	8.41×10^{-10}	0.031	0
Brain Nucleus accumbens (basal ganglia)	2.19×10^{-9}	1.45×10^{-9}	0.066	0
Brain Putamen (basal ganglia)	7.54×10^{-10}	1.52×10^{-9}	0.310	0
Brain Caudate (basal ganglia)	-4.57×10^{-11}	1.40×10^{-9}	0.513	0
Brain Amygdala	-5.90×10^{-10}	9.80×10^{-10}	0.726	0
Brain Spinal cord (cervical c-1)	-7.30×10^{-10}	9.46×10^{-10}	0.780	0
Brain Hypothalamus	-1.22×10^{-9}	8.42×10^{-10}	0.927	0
Brain Hippocampus	-1.57×10^{-9}	8.42×10^{-10}	0.969	0
Brain Substantia nigra	-2.18×10^{-9}	8.54×10^{-10}	0.995	0

Multiple comparisons were controlled for using a Bonferroni correction across the 13 annotations (α = 0.003846154) where 1 = Yes; 0 = No.

Supplementary Table 15. Showing the enrichment of the MTAG analysis of household income for 3 brain cell types taken from Cahoy et al. (2008).

Brain Cell Type	Coefficient	Coefficient SE	Coefficient P-value	Significant
Neuron	4.30×10^{-9}	1.09×10^{-9}	3.95×10^{-5}	1
Oligodendrocyte	-4.24×10^{-10}	1.09×10^{-9}	0.651	0
Astrocyte	-6.32×10^{-10}	9.61×10^{-10}	0.745	0

Multiple comparisons were controlled for using a Bonferroni correction across the 3 annotations (α = 0.01666667) where 1 = Yes; 0 = No.

Supplementary Table 16. The source of the GWAS data sets used for genetic correlations and mtcojo

Phenotype	Consortium/Group	Reference	No. of individuals in GWAS
Intelligence	CCACE	Hill et al.(2018)[14]	248,482
Educational Attainment	SSGAC	Lee et al. (2018)[20]	766,345
Social Deprivation	CCACE	Hill et al. (2016)[21]	112,005
Parents age at Death	-	Pilling et al. (2017)[22]	75,244
Bipolar disorder	Psychiatric Genetics Consortium (PGC)	Sklar et al.(2011)[23]	7,481 cases 9,250 controls
Schizophrenia	Psychiatric Genetics Consortium (PGC)	Schizophrenia working group (2014)[24]	36,989 cases 113,075 controls
MDD	Psychiatric Genetics Consortium (PGC)	Daly et al. (2013)[25]	9,240 cases 9,519 controls
Anorexia nervosa	Genetic Consortium for Anorexia Nervosa (GCAN)	Boraska et al. (2014)[26]	2,907 cases 14,860 controls
ADHD	Psychiatric Genetics Consortium (PGC)	Demontis et al. (2019)[27]	19,099 cases 34,194 controls
ASD	Psychiatric Genetics Consortium (PGC)	Cross-Disorder Group of the Psychiatric Genomics Consortium (2013)[28]	10,226
Coronary Artery Disease	CARDIoGRAM	Schunkert et al. (2011)[29]	22,233 cases 64,762 controls
Type 2 diabetes	DIAGRAM	Morris et al. (2012)[30]	12,171 cases 56,862 controls
Obesity	GIANT	Berndt et al. (2013)[31]	263,407
Alzheimer's disease	CCACE	Marioni et al. (2018)[32]	388,324
Self-Rated Health	CCACE	Harris et al. (2016)[33]	111,749
Smoking (EverVsNever)	Tobacco and Genetics Consortium	Furberg et al. (2010)[34]	74,053
Tiredness	CCACE	Deary et al. (2017)[35]	108,976
FEV1	Spirometer	Artigas et al. (2015)[36]	38,199
Subjective Wellbeing	SSGAC	Okbay et al. (2015)[37]	193,397
BMI	GIANT	Locke et al. (2015)[38]	339,224
Height	GIANT	Wood et al. (2014)[39]	253,288
Head Circumference	EGG	Taal et al. (2012)[40]	10,678
Chronotype	-	Jones et al. (2016)[41]	128,266
Sleep Duration	-	Jones et al. (2016)[41]	128,266
Age at First Birth	SSGAC	Barban et al. (2016)[42]	222,037
Number of Children	SSGAC	Barban et al. (2016)[42]	318,863
Neuroticism (As measured using a general factor of Neuroticism)	CCACE	Hill et al. (2019)[43]	270,059
Anxiety/Tension (Special factor of Neuroticism)	CCACE	Hill et al. (2019)[43]	270,059
Worry/Vulnerability (Special factor of Neuroticism)	CCACE	Hill et al. (2019)[43]	270,059

Chromosome 1

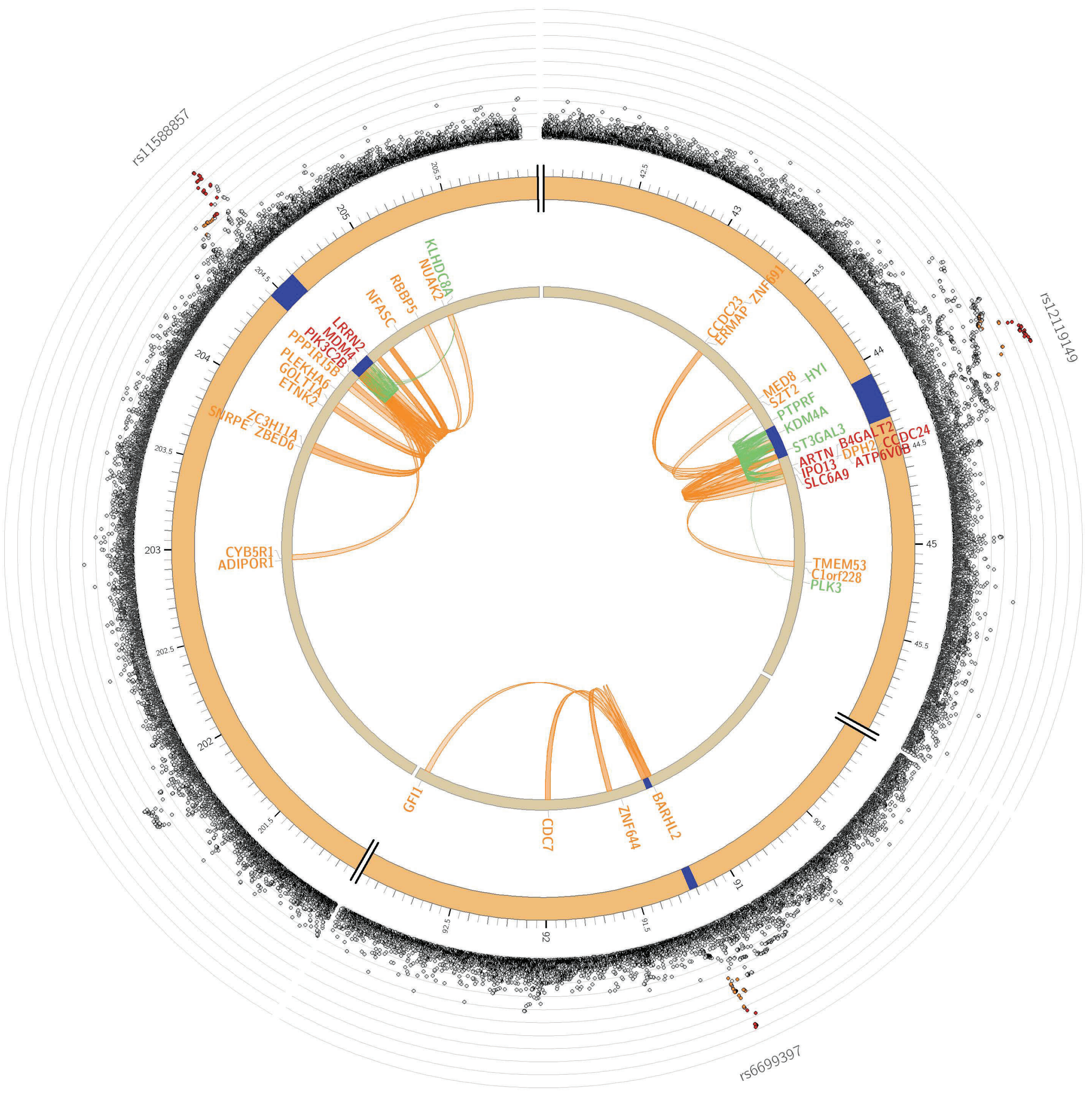

Chromosome 2

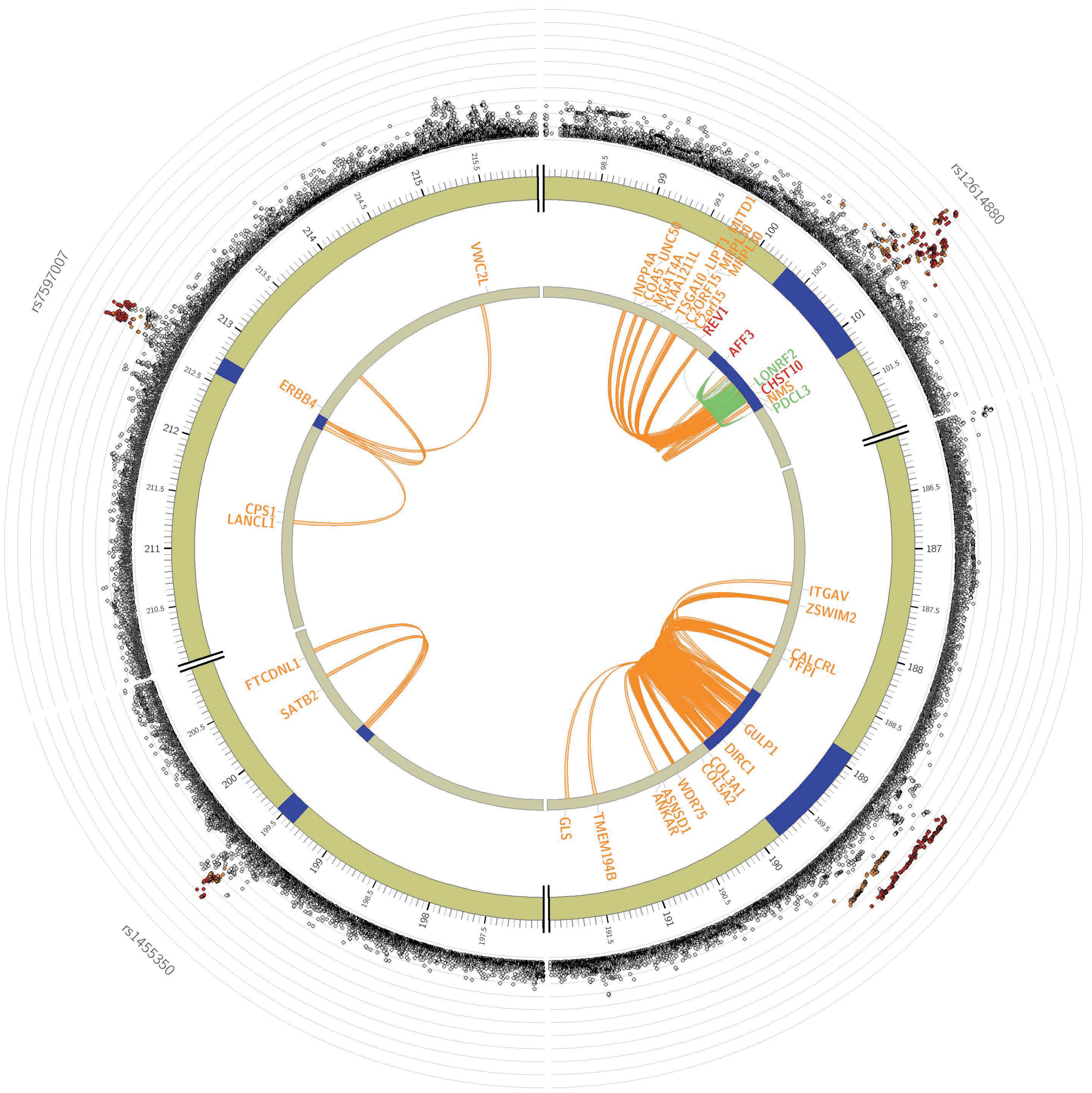

Chromosome 3

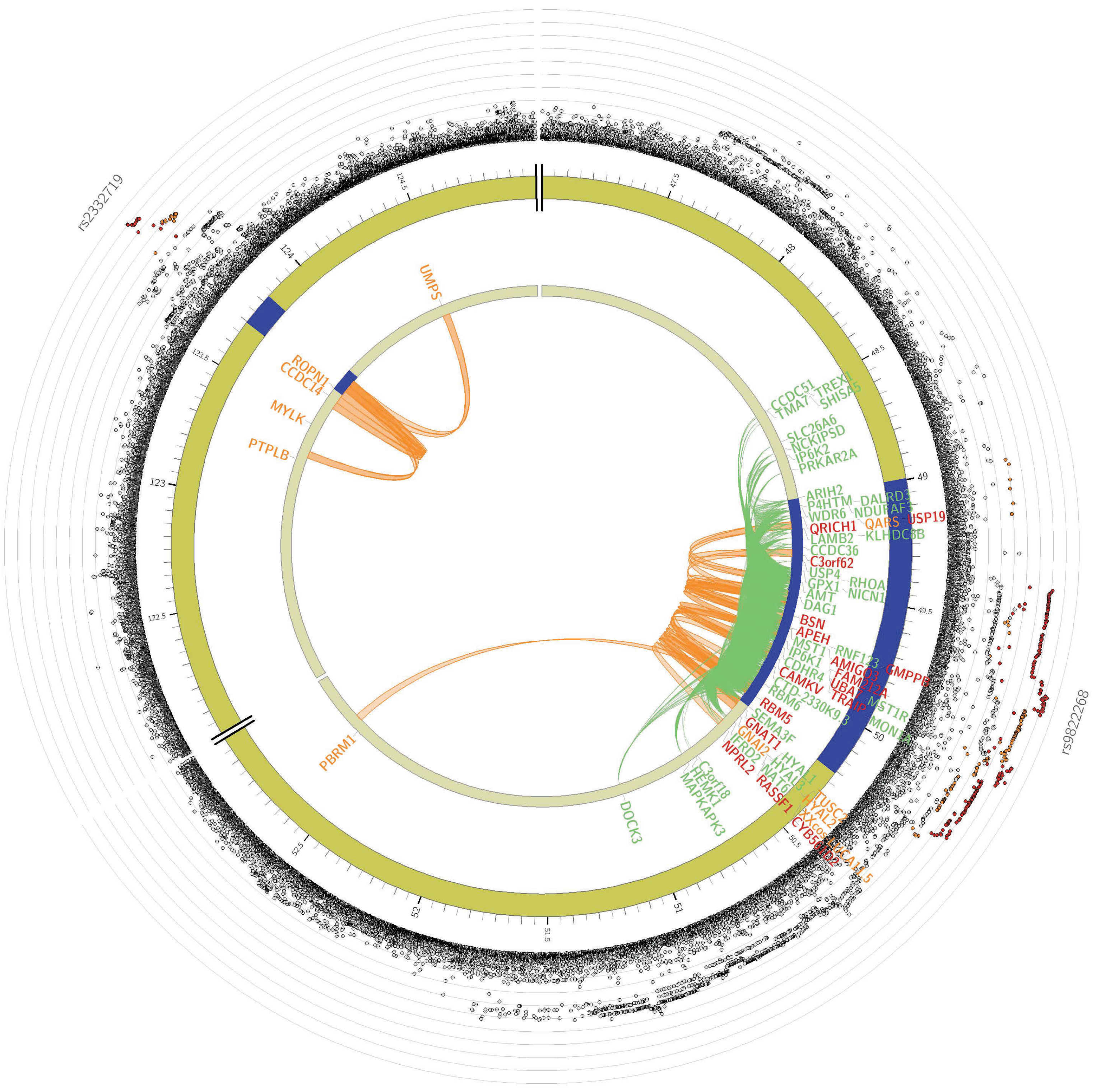

Chromosome 4

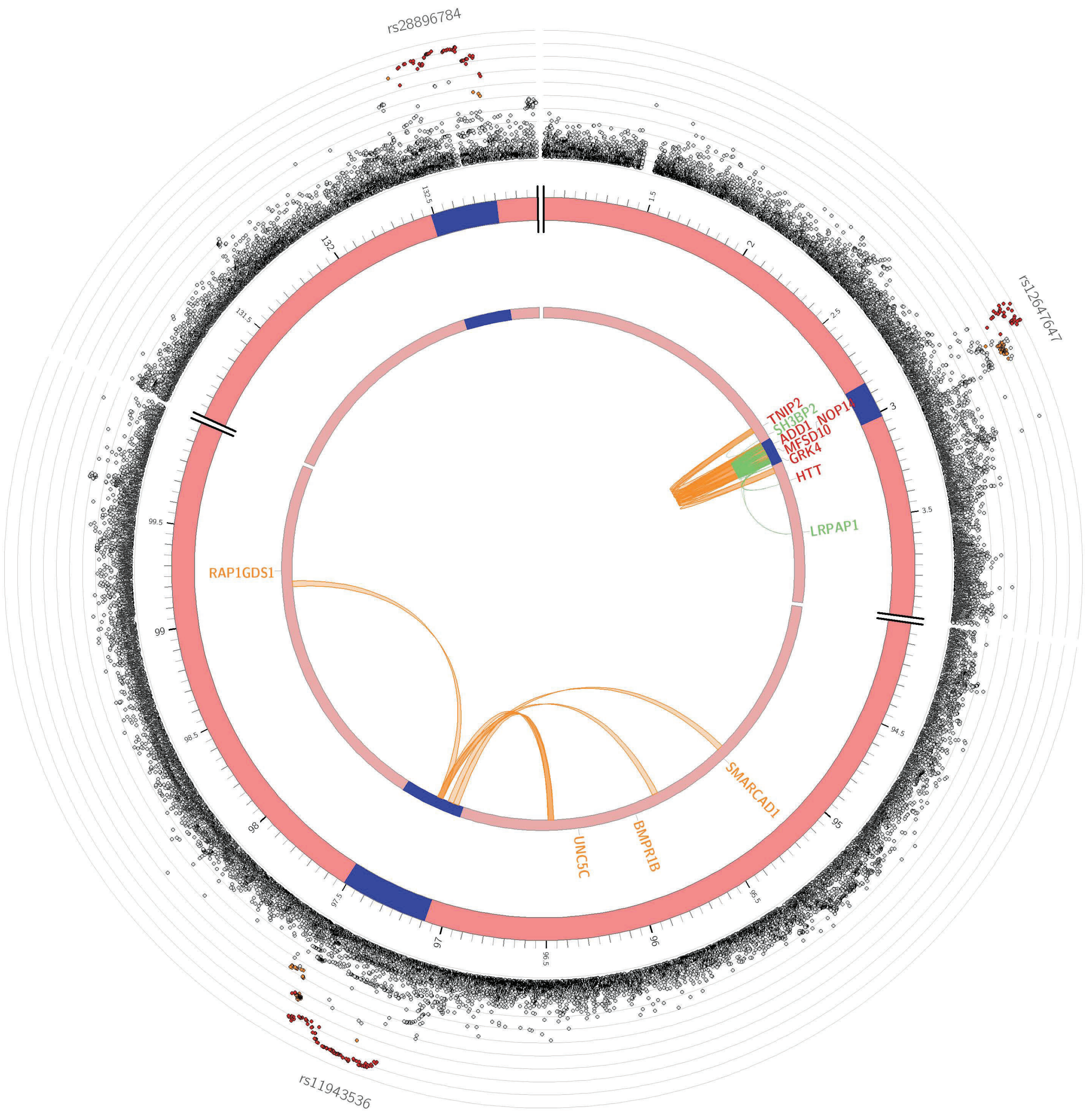

Chromosome 5

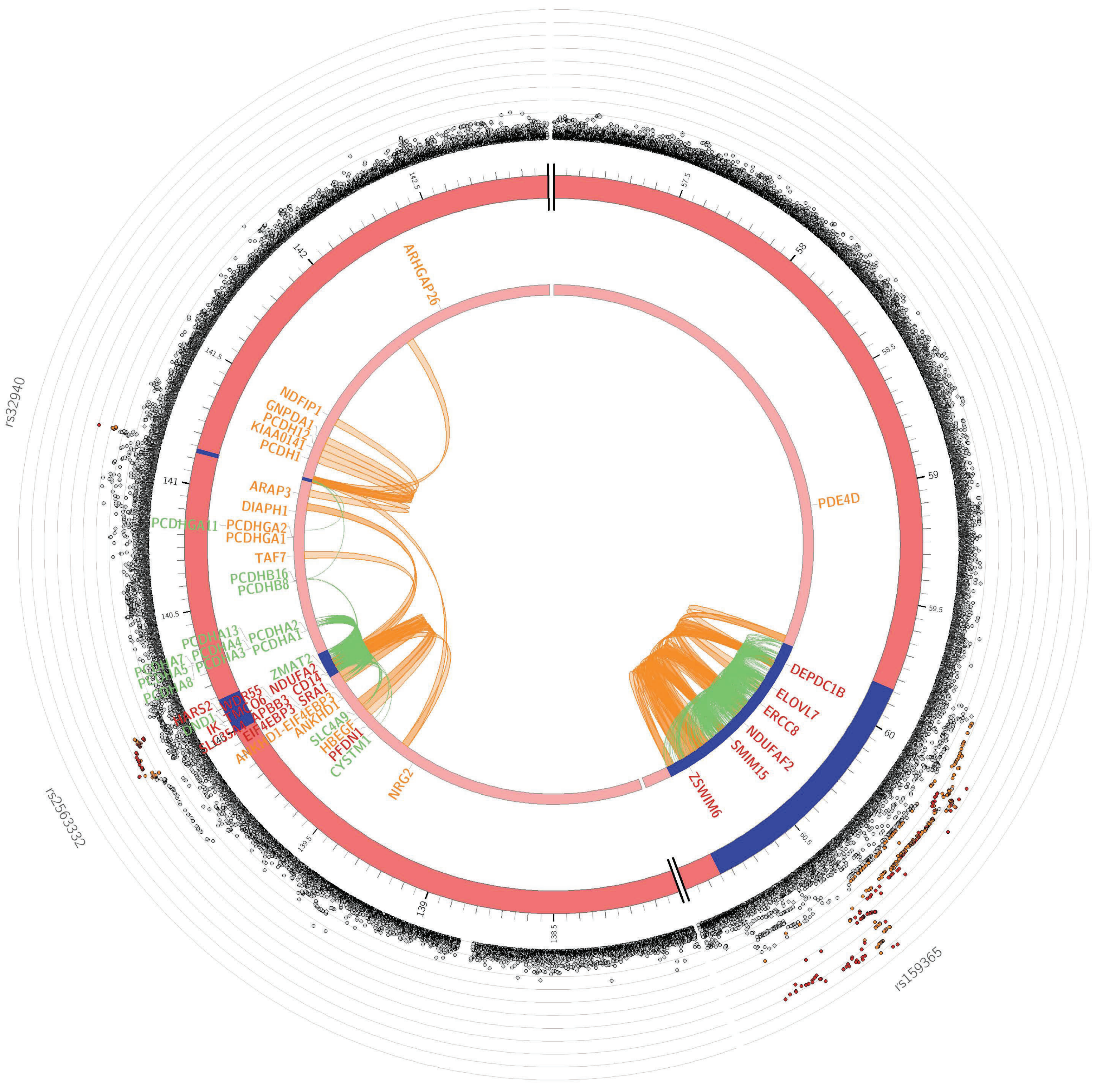

Chromosome 6

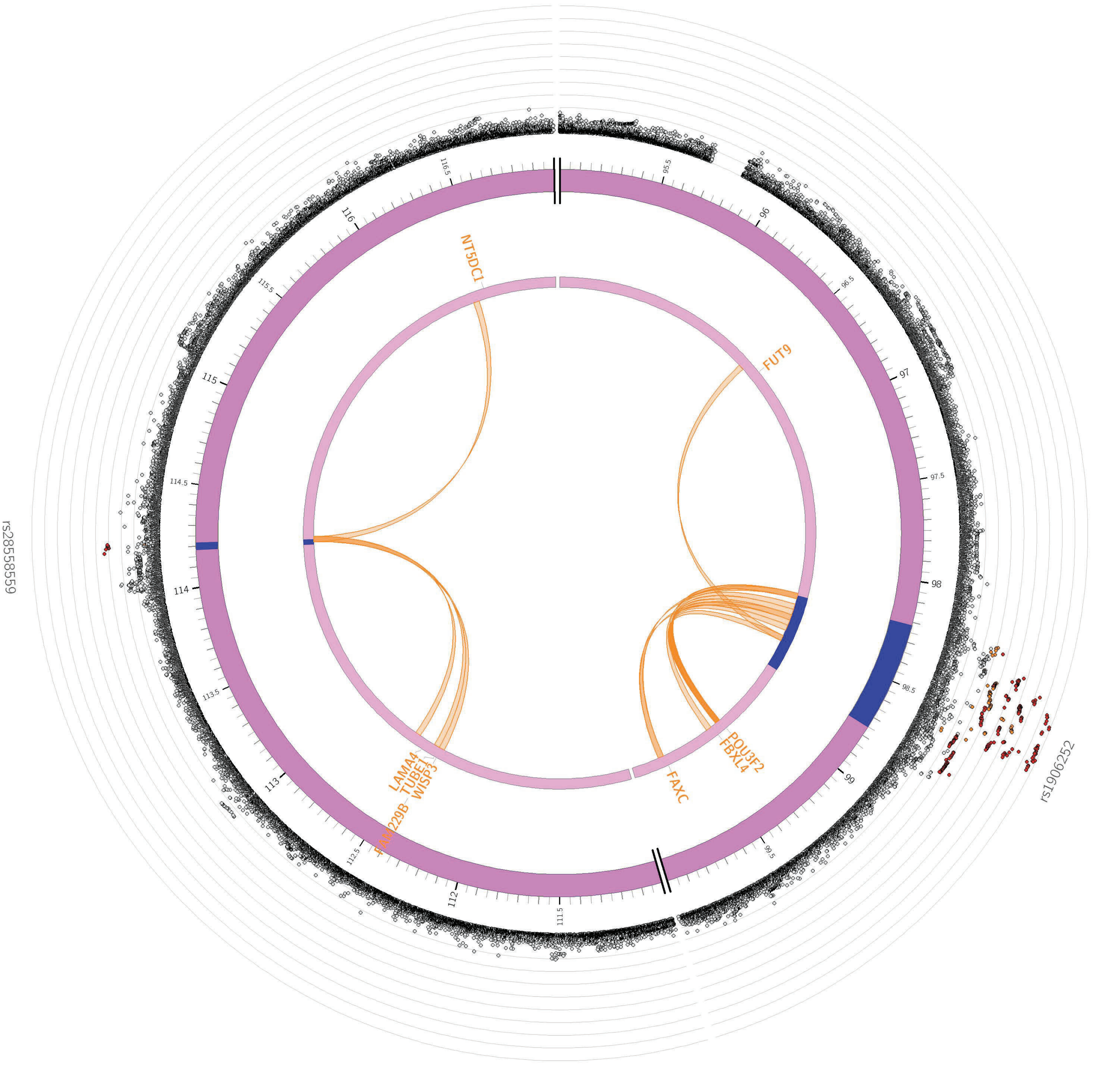

Chromosome 7

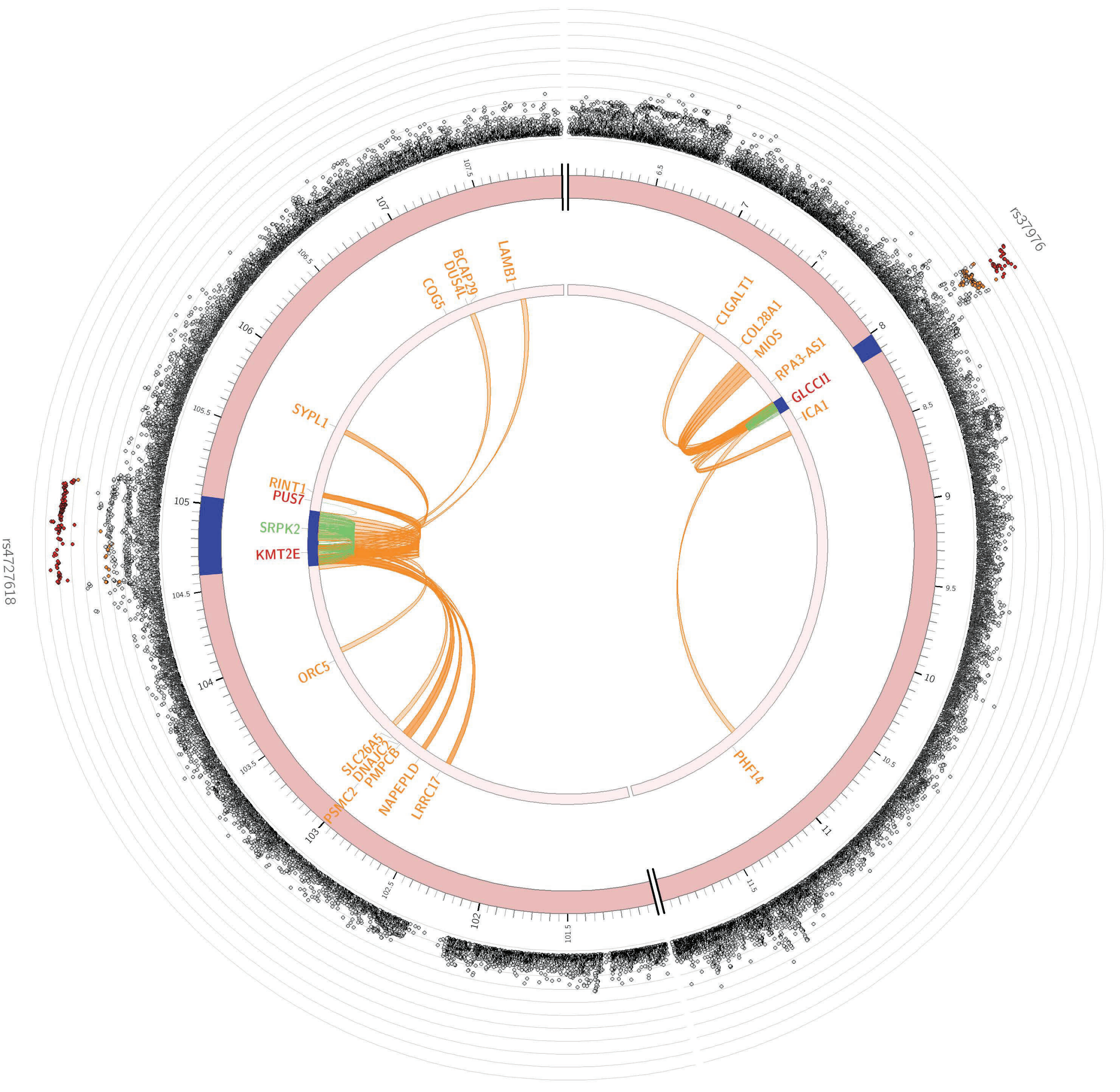

Chromosome 9

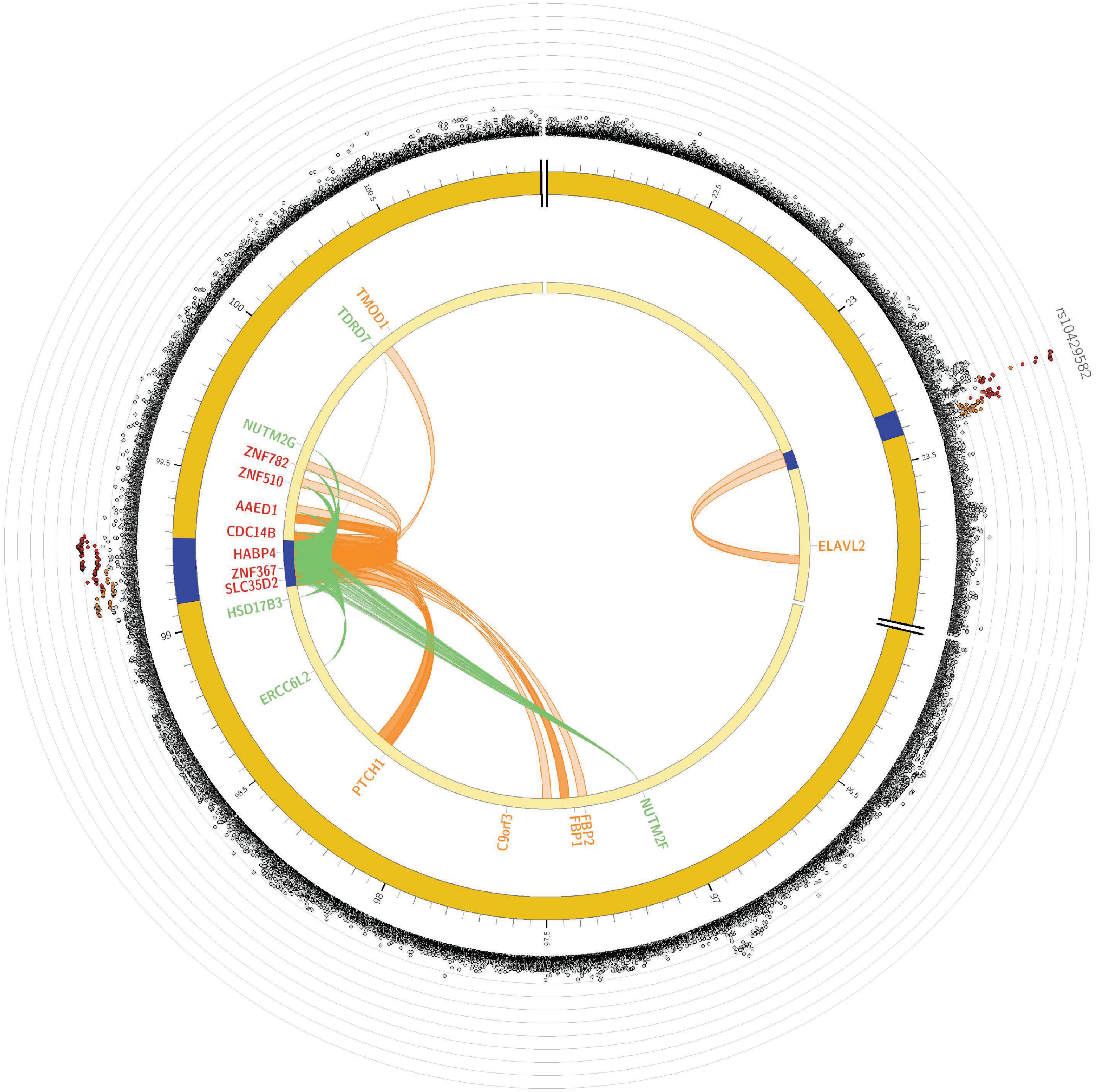

Chromosome 13

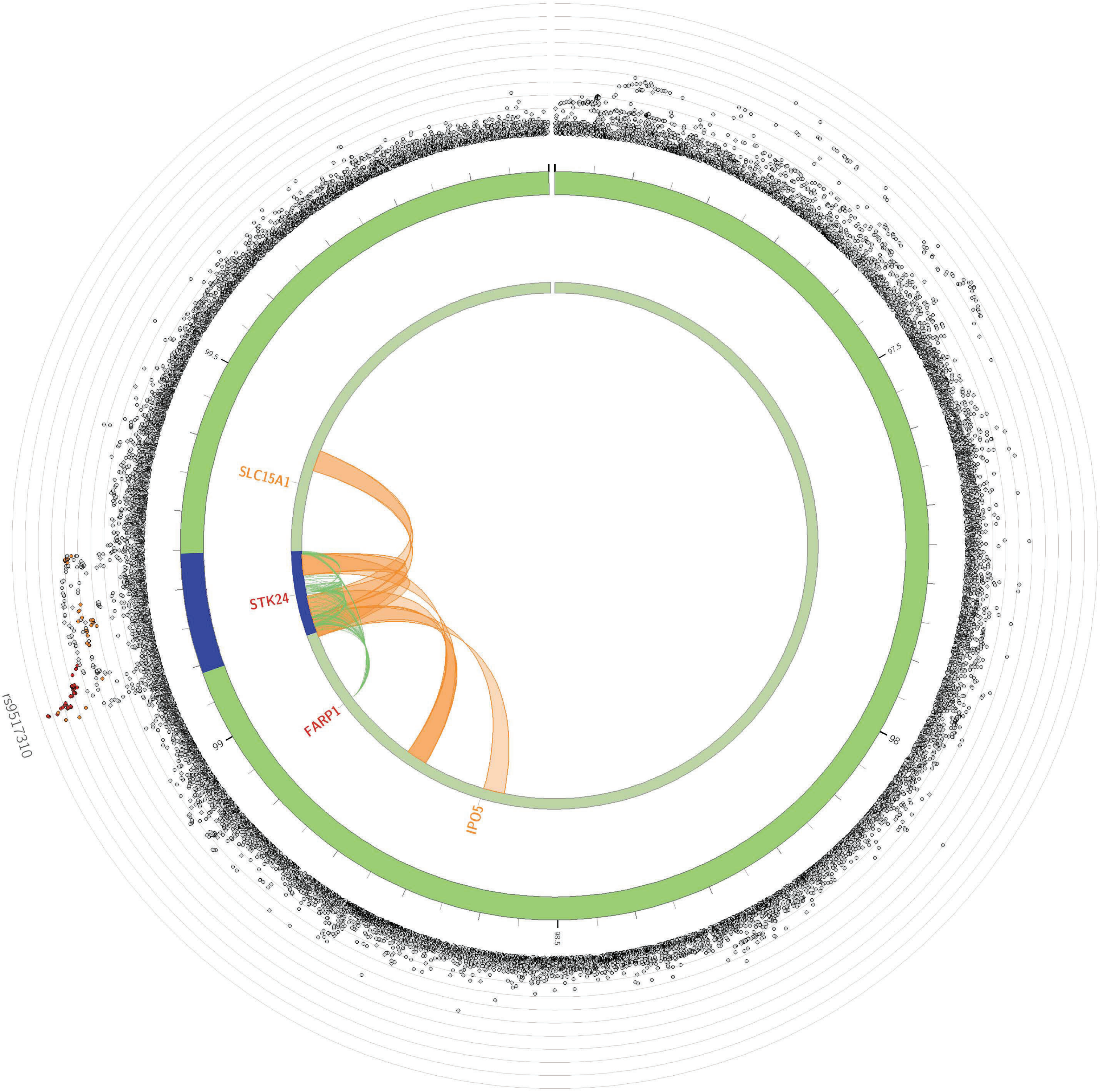

Chromosome 17

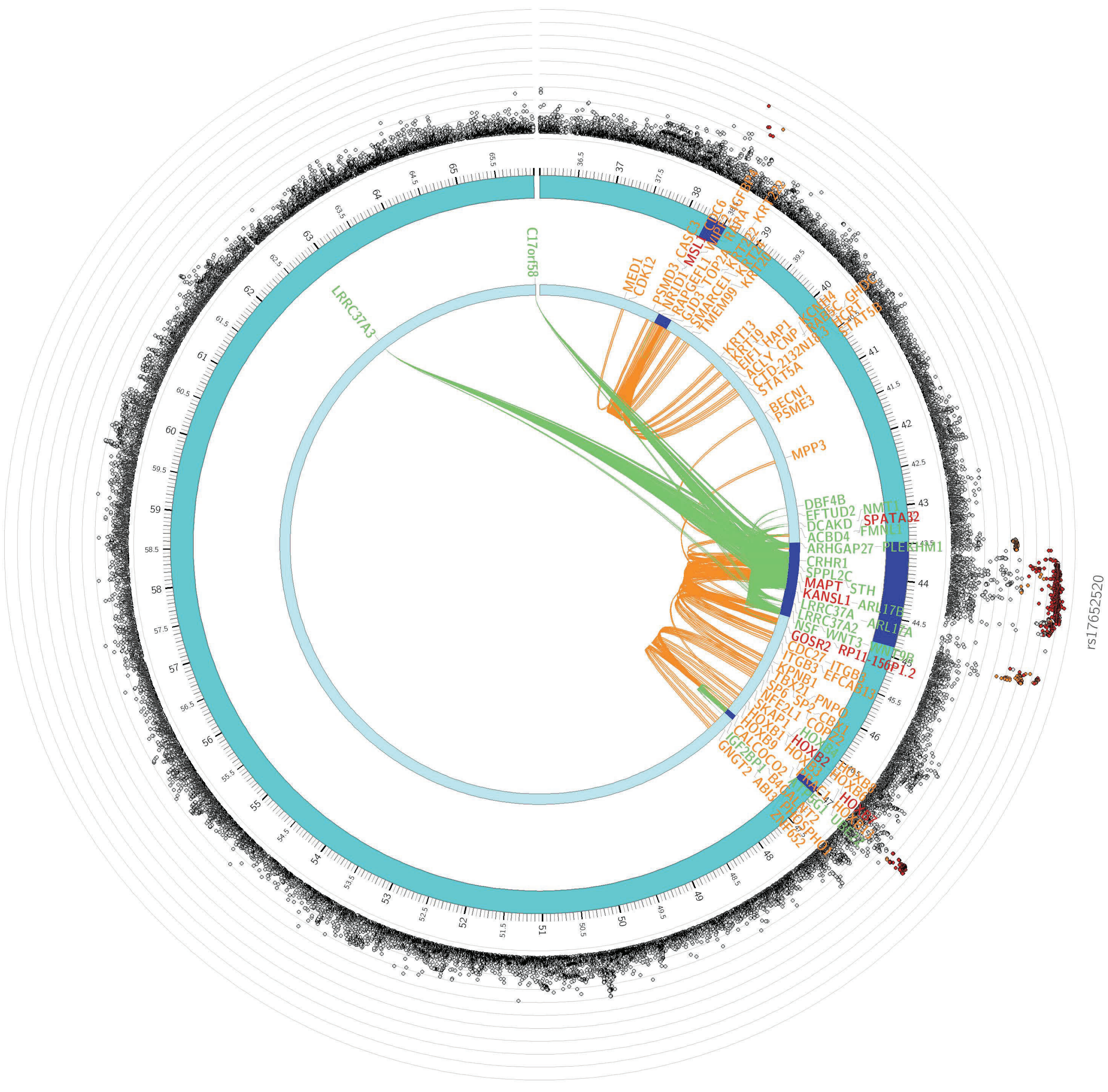

Chromosome 18

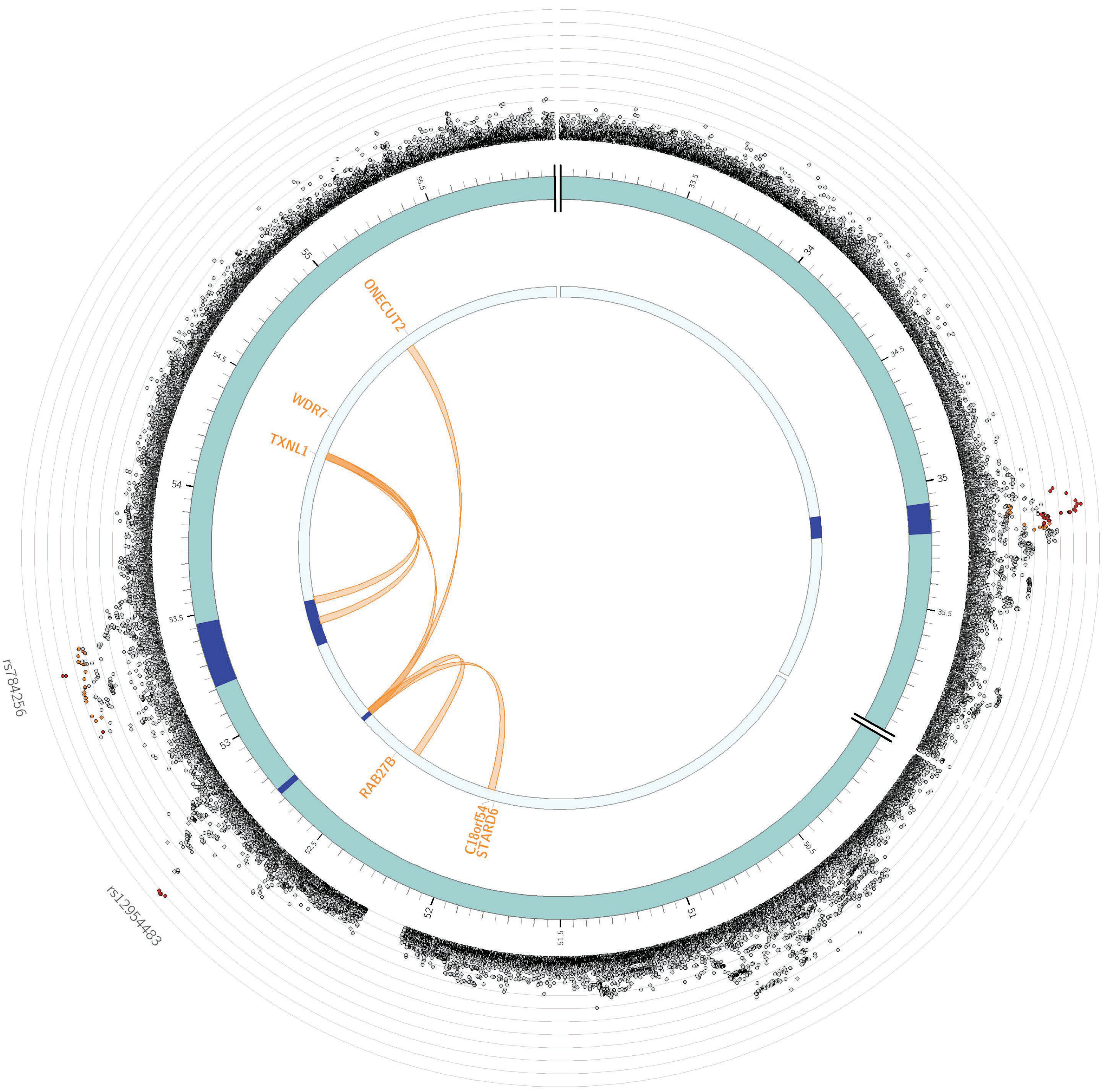

Chromosome 19

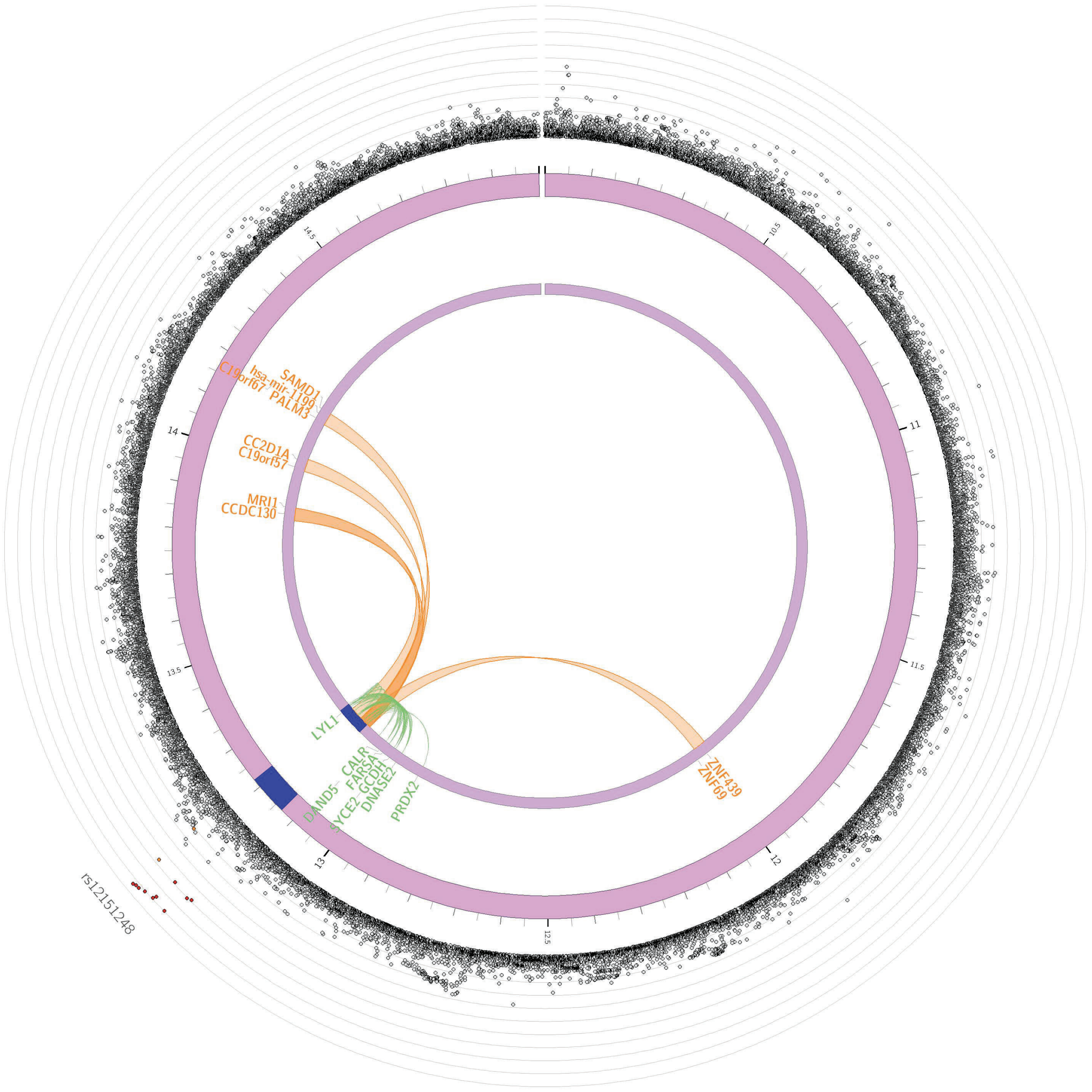

Chromosome 20

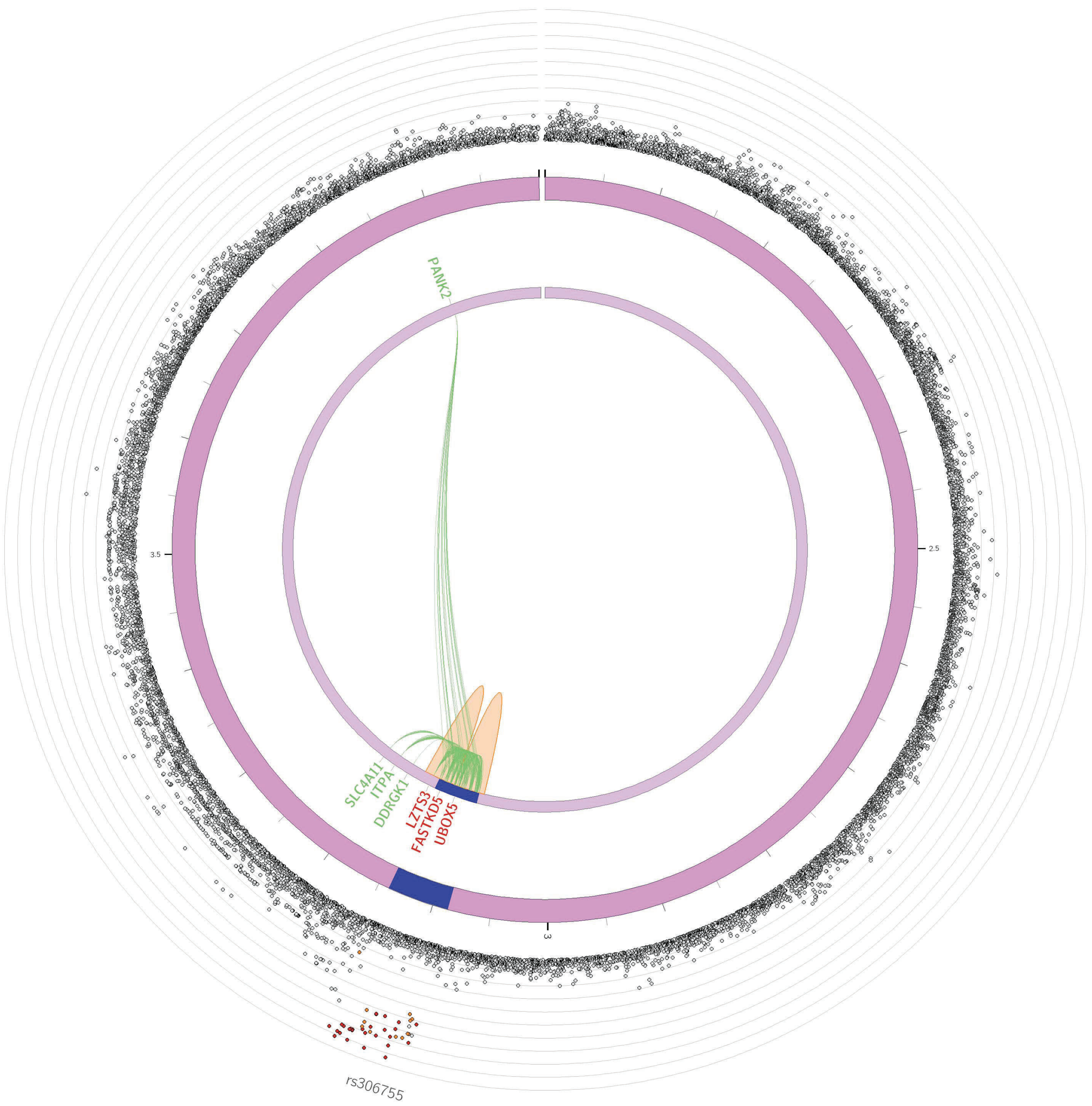

Supplementary Figure 1. Circos plots by chromosome illustrating genome-wide significant loci associated with income (Supplementary Figure 1. The most outer layer shows the Manhattan plot and only SNPs where P <0.05 are shown. Each of the SNPs in the genomic risk loci are colour coded indicating the maximum r^2 with one of the independent significant SNPs in the locus with red indicating the highest r^2 and blue the lowest r^2(red r^2>0.8, orange r^2>0.6, green r^2>0.4, and blue r^2>0.2). SNPs shown in grey are not in LD with any of the genome wide significant SNPs. The rsID of the most significant lead SNP in each loci is shown. The second layer is the chromosomal ring with the independent genomic risk loci highlighted in blue. Next, the genes mapped by chromatin interactions or eQTLs are displayed. Genes mapped using chromatin interactions the gene is displayed in orange, with genes mapped by eQTL shown in green. Genes that are displayed in red are those mapped using both chromatin interactions and eQTLs. Chromatin interaction links (coloured orange for chromatin interactions and green for eQTLs are displayed.

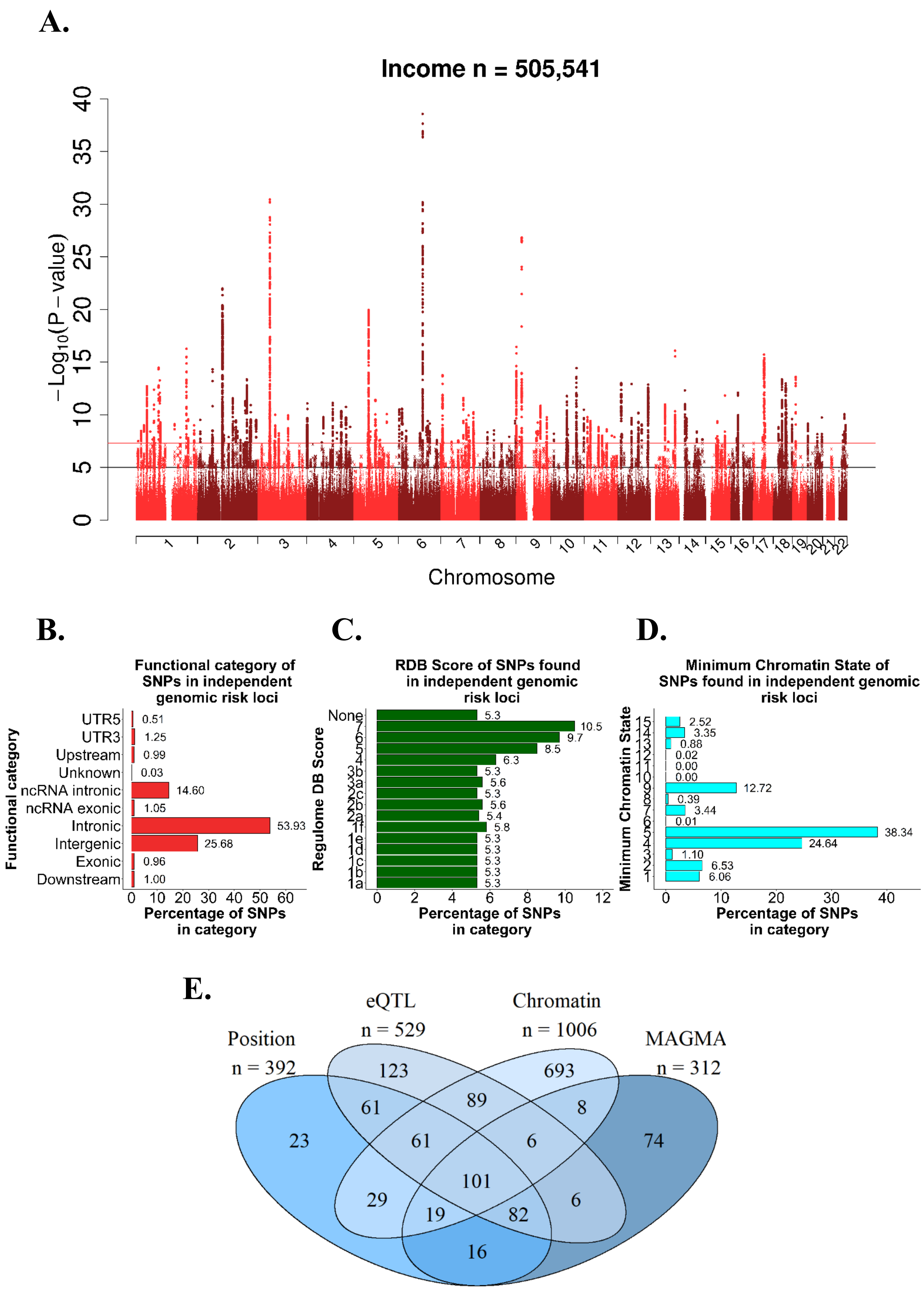

A.
Income n = 505,541
−Log10(P − value)
Chromosome
B.
Functional category of SNPs in independent genomic risk loci
UTR5 0.51
UTR3 1.25
Upstream 0.99
Unknown 0.03
ncRNA intronic 14.60
ncRNA exonic 1.05
Intronic 53.93
Intergenic 25.68
Exonic 0.96
Downstream 1.00
Functional category
Percentage of SNPs in category
C.
RDB Score of SNPs found in independent genomic risk loci
None 5.3
7 10.5
6 9.7
5 8.5
4 6.3
3b 5.3
3a 5.6
2c 5.3
2b 5.6
2a 5.4
1f 5.8
1e 5.3
1d 5.3
1c 5.3
1b 5.3
1a 5.3
Regulome DB Score
Percentage of SNPs in category
D.
Minimum Chromatin State of SNPs found in independent genomic risk loci
15 2.52
14 3.35
13 0.88
12 0.02
11 0.00
10 0.00
9 12.72
8 0.39
7 3.44
6 0.01
5 38.34
4 24.64
3 1.10
2 6.53
1 6.06
Minimum Chromatin State
Percentage of SNPs in category
E.
Position n = 392
eQTL n = 529
Chromatin n = 1006
MAGMA n = 312
123
693
61
89
8
23
61
6
74
101
29
19
82
6
16

Supplementary Figure 2A. Manhattan plot for MTAG derived income phenotype; $-\log_{10}$ transformed P-values for each SNP are plotted against chromosomal location. The red line indicates genome-wide significance and the black line indicates suggestive associations. **Supplementary Figure 2B**. Functional annotation carried out on the independent genomic loci identified. The percentage of SNPs found in each of the nine functional categories is listed. **Supplementary Figure 2C**. The percentage of SNPs from the independent genomic loci that fell into each of the Regulome DB scores categories. A lower score indicates greater evidence for that SNPs involvement in gene regulation. **Supplementary Figure 2D**. The percentage of SNPs within the independent genomic loci plotted against the minimum chromatic state for 127 tissue/cell types. **Supplementary Figure 2E**. Venn diagram illustrating the overlap of the genes implicated using positional mapping, eQTL mapping, chromatin interaction mapping, that was conducted on the independent significant loci identified in the SNP-based GWAS. Also shown is how these implicated genes overlap with those identified using the gene-based statistics derived using MAGMA.

Supplementary References

1. Di Angelantonio, E. *et al.* Efficiency and safety of varying the frequency of whole blood donation (INTERVAL): a randomised trial of 45 000 donors. *The Lancet* **390**, 2360-2371 (2017).
2. Moore, C. *et al.* The INTERVAL trial to determine whether intervals between blood donations can be safely and acceptably decreased to optimise blood supply: study protocol for a randomised controlled trial. *Trials* **15**, 363 (2014).
3. Purcell, S. *et al.* PLINK: a tool set for whole-genome association and population-based linkage analyses. *The American Journal of Human Genetics* **81**, 559-575 (2007).
4. The Genomes Project, C. *et al.* A global reference for human genetic variation. *Nature* **526**, 68 (2015).
5. Jun, G. *et al.* Detecting and Estimating Contamination of Human DNA Samples in Sequencing and Array-Based Genotype Data. *The American Journal of Human Genetics* **91**, 839-848 (2012).
6. Astle, W.J. *et al.* The Allelic Landscape of Human Blood Cell Trait Variation and Links to Common Complex Disease. *Cell* **167**, 1415-1429.e19 (2016).
7. Gallacher, J. *et al.* A Platform for the Remote Conduct of Gene-Environment Interaction Studies. *PLOS ONE* **8**, e54331 (2013).
8. Manichaikul, A. *et al.* Robust relationship inference in genome-wide association studies. *Bioinformatics* **26**, 2867-2873 (2010).
9. Loh, P.-R. *et al.* Efficient Bayesian mixed-model analysis increases association power in large cohorts. *Nature Genetics* **47**, 284 (2015).
10. Sniekers, S. *et al.* Genome-wide association meta-analysis of 78,308 individuals identifies new loci and genes influencing human intelligence. *Nature Genetics* **49**, 1107 (2017).
11. Willer, C.J., Li, Y. & Abecasis, G.R. METAL: fast and efficient meta-analysis of genomewide association scans. *Bioinformatics* **26**, 2190-2191 (2010).
12. Zhu, Z. *et al.* Integration of summary data from GWAS and eQTL studies predicts complex trait gene targets. *Nature Genetics* **48**, 481 (2016).
13. Watanabe, K., Taskesen, E., Bochoven, A. & Posthuma, D. Functional mapping and annotation of genetic associations with FUMA. *Nature communications* **8**, 1826 (2017).
14. Hill, W. *et al.* A combined analysis of genetically correlated traits identifies 187 loci and a role for neurogenesis and myelination in intelligence. *Molecular psychiatry*, 1 (2018).
15. Hill, W.D., Weiss, A., McIntosh, A.M., Gale, C.R. & Deary, I.J. Genetic contribution to two factors of neuroticism is associated with affluence, better health, and longer life. *bioRxiv* (2017).
16. Solovieff, N., Cotsapas, C., Lee, P.H., Purcell, S.M. & Smoller, J.W. Pleiotropy in complex traits: challenges and strategies. *Nature reviews. Genetics* **14**, 483 (2013).
17. Hyytinen, A., Ilmakunnas, P., Johansson, E. & Toivanen, O. Heritability of lifetime earnings. *The Journal of Economic Inequality* **17**, 319-335 (2019).
18. Bulik-Sullivan, B.K. *et al.* LD Score regression distinguishes confounding from polygenicity in genome-wide association studies. *Nature genetics* **47**, 291-295 (2015).
19. Leake, J. Scientists find 24 ‘golden’ genes that help you get rich. *The Times*, Retrieved from https://www.thetimes.co.uk (2019).
20. Lee, J.J. *et al.* Gene discovery and polygenic prediction from a genome-wide association study of educational attainment in 1.1 million individuals. *Nature Genetics* **50**, 1112-1121 (2018).
21. Hill, W.D. *et al.* Molecular genetic contributions to social deprivation and household income in UK Biobank. *Current Biology* **26**, 3083-3089 (2016).
22. Pilling, L.C. *et al.* Human longevity: 25 genetic loci associated in 389,166 UK biobank participants. *Aging* **9**, 2504-2520 (2017).
23. Sklar, P. *et al.* Large-scale genome-wide association analysis of bipolar disorder identifies a new susceptibility locus near ODZ4. *Nature Genetics* **43**, 977-983 (2011).
24. Schizophrenia Working Group of the Psychiatric Genomics, C. *et al.* Biological insights from 108 schizophrenia-associated genetic loci. *Nature* **511**, 421 (2014).

25. Daly, J. *et al.* A mega-analysis of genome-wide association studies for major depressive disorder. *Mol Psychiatry.* **18**(2013).
26. Boraska, V. *et al.* A genome-wide association study of anorexia nervosa. *Molecular Psychiatry* **19**, 1085-1094 (2014).
27. Demontis, D. *et al.* Discovery of the first genome-wide significant risk loci for attention deficit/hyperactivity disorder. *Nature Genetics* **51**, 63-75 (2019).
28. Consortium, C.-D.G.o.t.P.G. Identification of risk loci with shared effects on five major psychiatric disorders: a genome-wide analysis. *The Lancet* **381**, 1371-1379 (2013).
29. Schunkert, H. *et al.* Large-scale association analysis identifies 13 new susceptibility loci for coronary artery disease. *Nature Genetics* **43**, 333-338 (2011).
30. Morris, A.P. *et al.* Large-scale association analysis provides insights into the genetic architecture and pathophysiology of type 2 diabetes. *Nature Genetics* **44**, 981-990 (2012).
31. Berndt, S.I. *et al.* Genome-wide meta-analysis identifies 11 new loci for anthropometric traits and provides insights into genetic architecture. *Nature Genetics* **45**, 501 (2013).
32. Marioni, R.E. *et al.* GWAS on family history of Alzheimer's disease. *Translational Psychiatry* **8**, 99 (2018).
33. Harris, S.E. *et al.* Molecular genetic contributions to self-rated health. *International Journal of Epidemiology* **46**, 994-1009 (2016).
34. Furberg, H. *et al.* Genome-wide meta-analyses identify multiple loci associated with smoking behavior. *Nature Genetics* **42**, 441-447 (2010).
35. Deary, V. *et al.* Genetic contributions to self-reported tiredness. *Molecular Psychiatry* **23**, 609-620 (2018).
36. Artigas, M.S. *et al.* Sixteen new lung function signals identified through 1000 Genomes Project reference panel imputation. *Nature Communications* **6**, 8658 (2015).
37. Okbay, A. *et al.* Genetic variants associated with subjective well-being, depressive symptoms, and neuroticism identified through genome-wide analyses. *Nature Genetics* **48**, 624 (2016).
38. Locke, A.E. *et al.* Genetic studies of body mass index yield new insights for obesity biology. *Nature* **518**, 197 (2015).
39. Wood, A.R. *et al.* Defining the role of common variation in the genomic and biological architecture of adult human height. *Nature Genetics* **46**, 1173 (2014).
40. Taal, H.R. *et al.* Common variants at 12q15 and 12q24 are associated with infant head circumference. *Nature Genetics* **44**, 532-538 (2012).
41. Jones, S.E. *et al.* Genome-Wide Association Analyses in 128,266 Individuals Identifies New Morningness and Sleep Duration Loci. *PLOS Genetics* **12**, e1006125 (2016).
42. Barban, N. *et al.* Genome-wide analysis identifies 12 loci influencing human reproductive behavior. *Nature Genetics* **48**, 1462 (2016).
43. Hill, W.D. *et al.* Genetic contributions to two special factors of neuroticism are associated with affluence, higher intelligence, better health, and longer life. *Molecular Psychiatry* (2019).
