# Supplementary material for: Genome-wide analysis identifies molecular systems and 149 genetic loci associated with income
Source: Nat Commun. 2019 Dec 16;10:5741. doi: 10.1038/s41467-019-13585-5 (PMC6915786; doi:10.1038/s41467-019-13585-5)
Supplement: Supplementary file 3 — Description of Additional Supplementary Files [file 41467_2019_13585_MOESM3_ESM.pdf]

Description of Additional Supplementary Files.

File Name: Supplementary Data 1

Description: Independent lead SNPs and SNPs tagged within each identified genomic locus for household income. Nearest gene was defined based on ANNOVAR annotations showing the most deleterious annotation for each of the tagged SNPs. The functional consequence of genetic variation in each SNP was also defined using ANNOVAR. CADD scores describe the how deleterious this genetic variation in each of these SNPs is with higher numbers indicating a more deleterious SNP. RegulomeDB score (RDB score) provides a metric describing the biological evidence indicating that this SNP acts as a regulatory element. RDB scores range from 1a (the most evidence to 7 (the least evidence). minChrState is the minimum 15-core chromatin state across 127 tissue/cell types. A score <8 indicates an open chromatin state.

File Name: Supplementary Data 2

Description: Note: Overview of all independent genomic loci identified for household income in the current study, and the two loci identified in Hill et al. (2016). Columns A-G refer to data from the current study, whereas columns H-J are derived from Hill et al. (2016). Genomic locus is the independent genetic locus identified in the analysis of household income (see Supplementary Table 1); CHR pertains to the chromosome number on which the locus is located on; Start/End is the start/end position, in base pairs of the locus in the current study (column C-D) and in the Hill et al. (2016) study (column I-J); rsID rs number of the top lead SNP located in the given locus; BP refers to the base pair position of the lead SNP in the current study; Located in Hill et al. (2016) locus shows whether or not one of the lead SNPs in the locus is located in the locus as reported by Hill et al (2016); Overlap indicates whether or not the locus identified in the current study physically overlaps with a locus reported by Hill et al. (2016). Novel indicates that the loci identified in the current study are novel and have not been previously associated with income. For columns G, K-L 0 = No; 1 = Yes.

File Name: Supplementary Data 3

Description: Lead SNPs and tagged SNPs for household income that have previously been identified at $P < 9 \times 10^{-6}$ in GWAS of diseases and traits listed in the GWAS catalog 2018-02-06 (www.ebi.ac.uk/gwas/).

File Name: Supplementary Data 4

Description: Genes implicated by positional mapping, eQTL mapping, and/ or chromatin interaction mapping of significant SNPs using household income.

Loci for genes where their position in the reference genome spanned two genomic risk loci are listed as Genomic Locus1: Genomic Locus2. Prob loss of function = probability of loss of function mutation intolerance (higher scores indicate higher probability); Positional Mapping #SNPs = the number of SNPs mapped to this gene by positional mapping, Positional Mapping Max CADD = Combined Annotation-Dependent Depletion score (a high score indicates a more deleterious effect), ncRVIS = non-coding residual variation intolerance score. The higher the score the more intolerant to noncoding variants the gene is, eqtl Mapping #SNPs = number of SNPs mapped to this gene by expression

quantitative trait locus mapping, eqtl Mapping min P = the minimum P value of the eQTL association for the mapped gene, eqtl Mapping min Q = the minimum false discovery rate q value of the eQTL association for the mapped gene, eqtl Mapping Tissue = the tissues that the significant eQTL association was observed in, eqtl Direction = the direction of association of the eQTL SNP with expression levels (up or downregulation), Chromatin Interaction Mapping = chromatin interaction mapping Chromatin Interaction Mapping = the tissues that the significant chromatin interaction was observed in, min GWAS P = the minimum GWAS P value of each SNP implicating the gene, Independent significant SNPs = SNPs with significant GWAS p-values from which the gene was mapped, Gene Type = Status of the gene from Ensembl.

File Name: Supplementary Data 5

Description: Chromatin interaction regions linking significant GWAS loci to genes using household income.

Interaction regions (Interaction region 1 (BP) - Interaction region 2 (BP)) include one region overlapping one or more GWAS SNP in an enhancer, and one region overlapping a gene promoter region. GWAS SNPs in interaction region : rsID of candidate SNPs which are overlapping with the region 1. Genes within interaction region : ENSG ID of genes whose promoter regions are overlapped with region 2.

File Name: Supplementary Data 6

Description: MAGMA gene based association for household income. Significant = 1, indicates genes that withstood Bonferroni correction (2.662 E-6).

File Name: Supplementary Data 7

Description: MAGMA gene-set analysis for household income. No gene sets withstood Bonferroni correction (4.59E-6). Abbreviations: GO, Gene ontology; bp, biological functions; cc, cellular component; mp, molecular functions.

File Name: Supplementary Data 8

Description: MAGMA gene-property analysis for household income. Gene sets that withstood Bonferroni correction are highlighted in bold. 53 specific tissue types were created using gene expression data based on GTEx RNA-seq data. Significant = 1, indicates tissue groupings that withstood Bonferroni correction.

File Name: Supplementary Data 9

Description: Showing the fold enrichment for the functional groupings plus the additional boundaries, and peaks for heritability of income. A Bonferroni correction was used to control for multiple tests on the 57 functional categories ($\alpha = 0.000877193$). Significance indicates which functional categories and cell specific categories remained statistically significant following correction for multiple comparisons where 1 = Yes; 0 = No.

File Name: Supplementary Data 10

Description: Showing the enrichment of household income for 205 annotations based on gene expression. Multiple comparisons were controlled for using a false discovery rate ($\alpha = 0.005578054$) applied to all 205 annotations where 1 = Yes; 0 = No.

File Name: Supplementary Data 11

Description: Showing the enrichment of household income for 489 annotations based on chromatin. Multiple comparisons were controlled for using a false discovery rate ($\alpha = 0.003198245$) applied to all 489 annotations where 1 = Yes; 0 = No.

File Name: Supplementary Data 12

Description: Showing the genetic correlations performed using household income, household income conditioned on intelligence, intelligence, intelligence conditioned on household income, and education. Control for multiple comparisons was performed for each phenotype so 27 tests were controlled for using False Discovery Rate (FDR). Statistical significance 1 = yes, 0 = no.

File Name: Supplementary Data 13

Description: Independent lead SNPs and SNPs tagged within each identified genomic locus for the MTAG analysis of household income. Nearest gene was defined based on ANNOVAR annotations showing the most deleterious annotation for each of the tagged SNPs. The functional consequence of genetic variation in each SNP was also defined using ANNOVAR. CADD scores describe the how deleterious this genetic variation in each of these SNPs is with higher numbers indicating a more deleterious SNP. RegulomeDB score (RDB score) provides a metric describing the biological evidence indicating that this SNP acts as a regulatory element. RDB scores range from 1a (the most evidence to 7 (the least evidence). minChrState is the minimum 15-core chromatin state across 127 tissue/cell types. A score <8 indicates an open chromatin state.

File Name: Supplemental Data 14

Description: Overview of all independent genomic loci identified for the MTAG analysis of household income, and the analysis conducted on household income without the use of MTAG. Columns A-G refer to data from the MTAG analysis of income, whereas columns H-J are derived from the income analysis conducted without the use of MTAG. Genomic locus is the independent genetic locus identified in the MTAG analysis of household income (see Supplementary Table 1); CHR pertains to the chromosome number on which the locus is located on; Start/End is the start/end position, in base pairs of the locus in the current study (column C-D) and in the analysis of household income without the use of MTAG (column I-J); rsID rs number of the top lead SNP located in the given locus; BP refers to the base pair position of the lead SNP in the current study; Located in the analysis of household income without MTAG locus shows whether or not one of the lead SNPs in the locus is located in the locus as reported by in Supplementary Table 1; Overlap indicates whether or not the locus identified in the current study physically overlaps with a locus reported by without the use of MTAG). Novel indicates that the loci identified in the current study are novel and were not identified without the use of MTAG. For columns G, K-L 0 = No; 1 = Yes.

File Name: Supplementary Data 15

Description: Lead SNPs and tagged SNPs for MTAG analysis of household income that have previously been identified at P < 9 × 10-6 in GWAS of diseases and traits listed in the GWAS catalog 2018-02-06 (www.ebi.ac.uk/gwas/).

File Name: Supplementary Data 16

Description: Genes implicated by positional mapping, eQTL mapping, and/ or chromatin interaction mapping of significant SNPs using the MTAG analysis of household income.

Loci for genes where their position in the reference genome spanned two genomic risk loci are listed as Genomic Locus1: Genomic Locus2. Prob loss of function = probability of loss of function mutation intolerance (higher scores indicate higher probability); Positional Mapping #SNPs = the number of SNPs mapped to this gene by positional mapping, Positional Mapping Max CADD = Combined Annotation-Dependent Depletion score (a high score indicates a more deleterious effect), ncRVIS = non-coding residual variation intolerance score. The higher the score the more intolerant to noncoding variants the gene is, eqtl Mapping #SNPs = number of SNPs mapped to this gene by expression quantitative trait locus mapping, eqtl Mapping min P = the minimum P value of the eQTL association for the mapped gene, eqtl Mapping min Q = the minimum false discovery rate q value of the eQTL association for the mapped gene, eqtl Mapping Tissue = the tissues that the significant eQTL association was observed in, eqtl Direction = the direction of association of the eQTL SNP with expression levels (up or downregulation), Chromatin Interaction Mapping = chromatin interaction mapping Chromatin Interaction Mapping = the tissues that the significant chromatin interaction was observed in, min GWAS P = the minimum GWAS P value of each SNP implicating the gene, Independent significant SNPs = SNPs with significant GWAS p-values from which the gene was mapped, Gene Type = Status of the gene from Ensembl.

File Name: Supplementary Data 17

Description: Chromatin interaction regions linking significant GWAS loci to genes using the MTAG analysis of household income.

Interaction regions (Interaction region 1 (BP) - Interaction region 2 (BP)) include one region overlapping one or more GWAS SNP in an enhancer, and one region overlapping a gene promoter region. GWAS SNPs in interaction region: rsID of candidate SNPs which are overlapping with the region 1. Genes within interaction region: ENSG ID of genes whose promoter regions are overlapped with region 2.

File Name: Supplementary Data 18

Description: MAGMA gene based association for the MTAG analysis of household income. Significant = 1, indicates the genes that withstood Bonferroni correction (2.699E-6).

File Name: Supplementary Data 19

Description: MAGMA gene-set analysis for the MTAG analysis of household income. Significant = 1, indicates gene sets that withstood Bonferroni correction (4.59E-6). Abbreviations: GO, Gene ontology; bp, biological functions; cc, cellular component; mp, molecular functions.

File Name: Supplementary Data 20

Description: MAGMA gene-property analysis for MTAG analysis of household income. Gene sets that withstood Bonferroni correction are highlighted in bold. 53 specific tissue types were created using gene expression data based on GTEx RNA-seq data. Significant = 1, indicates tissue groupings that withstood Bonferroni correction.

File Name: Supplementary Data 21

Description: Showing the enrichment of the MTAG analysis of household income for 205 annotations based on gene expression. Multiple comparisons were controlled for using a false discovery rate ($\alpha = 0.005495216$) applied to all 205 annotations where 1 = Yes; 0 = No.

File Name: Supplementary Data 22

Description: Showing the enrichment of the MTAG analysis of household income for 489 annotations based on chromatin. Multiple comparisons were controlled for using a false discovery rate ($\alpha = 0.004303993$) applied to all 489 annotations where 1 = Yes; 0 = No.
